# A Cyclophilin Homology Domain-Independent Role for Nup358 in HIV-1 Infection

**DOI:** 10.1371/journal.ppat.1003969

**Published:** 2014-02-20

**Authors:** Anne M. Meehan, Dyana T. Saenz, Rebekah Guevera, James H. Morrison, Mary Peretz, Hind J. Fadel, Masakazu Hamada, Jan van Deursen, Eric M. Poeschla

**Affiliations:** 1 Department of Molecular Medicine, Mayo Clinic College of Medicine, Rochester, Minnesota, United States of America; 2 Department of Biochemistry & Molecular Biology, Mayo Clinic College of Medicine, Rochester, Minnesota, United States of America; Vanderbilt University School of Medicine, United States of America

## Abstract

The large nucleoporin Nup358/RanBP2 forms eight filaments that project from the nuclear pore into the cytoplasm where they function as docking platforms for nucleocytoplasmic transport receptors. RNAi screens have implicated Nup358 in the HIV-1 life cycle. The 164 C-terminal amino acids of this 3,224 amino acid protein are a cyclophilin homology domain (Nup358Cyp), which has potential to bind the HIV-1 capsid and regulate viral progress to integration. Here we examined the virological role of Nup358 in conditional knockout mouse cells and in RNAi-depleted human CD4+ T cells. Cre-mediated gene knockout was toxic and diminished HIV-1 infectivity. However, cellular health and HIV-1 susceptibility were coordinately preserved if, prior to gene inactivation, a transposon was used to express all of Nup358 or only the N-terminal 1340 amino acids that contain three FG repeats and a Ran-binding domain. HIV-1, but not N74D capsid-mutant HIV-1, was markedly sensitive to TNPO3 depletion, but they infected 1–1340 segment-complemented Nup358 knockout cells equivalently. Human and mouse CypA both rescued HIV-1 in CypA gene −/− Jurkat cells and TRIM-Nup358Cyp fusions derived from each species were equally antiviral; each also inhibited both WT and N74D virus. In the human CD4+ T cell line SupT1, abrupt Nup358 depletion reduced viral replication but stable Nup358-depleted cells replicated HIV-1 normally. Thus, human CD4+ T cells can accommodate to loss of Nup358 and preserve HIV-1 susceptibility. Experiments with cylosporine, viruses with capsids that do not bind cyclophilins, and growth arrest did not uncover viral dependency on the C-terminal domains of Nup358. Our data reinforce the virological importance of TNPO3 and show that Nup358 supports nuclear transport functions important for cellular homeostasis and for HIV-1 nuclear import. However, the results do not suggest direct roles for the Nup358 cyclophilin or SUMO E3 ligase domains in engaging the HIV-1 capsid prior to nuclear translocation.

## Author Summary

The purified cyclophilin homology domain (CHD) of Nup358/RanBP2 can interact with assembled HIV-1 capsids *in vitro*, which suggests that, in cells, the incoming virus core could be engaged functionally by the CHD prior to nucleopore traverse. Interpretations of Nup358 knockdowns have been complicated by toxicity due to globally altered cellular nucleocytoplasmic transport and by a lack of re-expression controls, which are difficult because of the protein's size. We present the first analyses of the HIV-1 life cycle in Nup358 knockout cells and in Nup358-depleted human CD4+ T cells, and the first detailed studies of domain requirements and re-expression controls. We find that the N-terminal portion containing three FG repeats rather than the C-terminal portion of Nup358 is sufficient to preserve HIV-1 infection susceptibility in dividing and non-dividing gene knockout cells. Mouse and human versions of CypA and Nup358Cyp are functionally equivalent and TNPO3 displays marked dependency factor activity in cells of both species. A human CD4+ T cell line can be stably deficient in Nup358 without loss of HIV-1 permissivity. The data support an important, though cyclophilin homology domain-independent, role for Nup358 in the HIV-1 life cycle and demonstrate conservation of HIV-1 early event pathways between human and mouse cells.

## Introduction

All lentiviruses infect non-dividing cells in the monocyte-macrophage lineage [1], [2]. This fundamental property has characterized this retroviral genus for over twelve million years [3] and is not limited to macrophages since lentiviral vectors readily transduce terminally differentiated or growth-arrested cells from diverse tissues [4], [5]. For HIV-1, proposed karyophilic mediators of pre-integration complex (PIC) nuclear pore transit have included signals in viral proteins (Matrix, Integrase, Vpr) as well as a short central plus strand discontinuity generated during reverse transcription [6]. Each has met with contradiction [7]–[12]. More recent evidence implicates the viral capsid [13]–[15].

Traffic between the cytosol and nucleus is gated by the nuclear pore complex (NPC), a 120 megadalton structure composed of multiple copies of more than 30 different nucleoporin proteins [16]. Nucleoporins with hydrophobic phenylalanine-glycine (FG) repeats line the central channel [17]. Transport receptors bind to import or export signals in macromolecules to mediate cargo translocation across the NPC via sequential low affinity FG nucleoporin interactions. Most transport receptors belong to a family of related proteins that includes the import receptor family karyopherin β (importin-β, transportin-1, transportin-3) and the export receptor CRM1 [18]. Some nuclear transport proteins and nucleoporins have recently been implicated by siRNA screens and other studies as HIV-1 dependency factors [19]–[29].

Nup358/RanBP2, hereafter designated by its initial name Nup358 [30], is the largest FG nucleoporin (358 kDa). The chief constituent of the eight NPC filaments that extend from the cytoplasmic face of the pore (each filament consists of one Nup358 molecule), it plays essential roles in nucleocytoplasmic trafficking and mitosis [31]. The most C-terminal segment of Nup358 is a cyclophilin homology domain (CHD). Cyclophilin proteins are peptidyl prolyl isomerases that catalyze *cis-trans* isomerization of peptide bonds at susceptible proline residues to facilitate correct protein folding. There are 16 human cyclophilin proteins [32]. Cyclophilin A (CypA) is a highly abundant cytoplasmic protein that binds to a conserved exposed loop in the HIV-1 capsid (CA) and facilitates HIV infection in human cells by still unclear mechanisms [33]–[35]. Although CypA is incorporated into HIV-1 virions [36], [37], viral replication phenotypes correlate with target cell CypA [38]–[41]. Cyclosporine (Cs) treatment or certain mutations in the cyclophilin binding loop of HIV-1 capsid (e.g., G89V), both of which abrogate CypA binding, impair HIV-1 infectivity. In contrast, in rhesus macaque cells CypA facilitates TRIM5alpha-mediated restriction of HIV-1 [41]–[43]. In CypA knockout (*PPIA* −/−) Jurkat CD4+ human T cells, wild type HIV-1 and G89V viruses are equally impaired and Cs does not have additive effect, which suggested that among the sixteen human cyclophilin domain-containing proteins, only CypA has a functionally relevant interaction with the HIV-1 capsid [44]. Recent evidence suggested that a primary function of CypA binding to CA is to prevent sensing of viral DNA in the cytosol and avert triggering of innate immune pathways [45], [46].

Nup358 scored as a hit in two large scale siRNA screens for HIV-1 dependency factors [19], [20]. Of potential interest in addition to the CHD, the C-terminal end of the protein also contains an active SUMO E3 ligase domain [47], [48]. There is evidence that some HIV-1 virion proteins are sumoylated [49], [50] and that sumoylation of certain cellular proteins precedes transport across the nuclear pore [31]. Nup358 also associates with and regulates interphase microtubules [51], which play a role in transit of the HIV-1 reverse transcription complex after nuclear entry [52]. To date, studies on the protein's role in the HIV life cycle have used RNAi to deplete it, often in HeLa or 293T cells [19], [20], [24], [26], [28], [29]. Nup358 knockdown was reported to impair infection of cells by HIV-1 in these studies and two observed altered integration site distributions after knockdown [28], [29]. Using purified proteins, Schaller et al. subsequently showed that the Nup358 CHD can bind to the HIV-1 CA N-terminal domain (NTD) in vitro [29]. A TRIM-Nup358Cyp fusion protein constructed to mimic a naturally occurring TRIM-Cyp protein [53] inhibited infection [29]. Certain HIV-1 viral capsid mutants, such as N74D, were not impaired by Nup358 knockdown in HeLa cells and interact poorly with Nup358Cyp in vitro; however they remained puzzlingly sensitive to TRIM-Nup358Cyp [29]. CypA interaction-abrogating capsid mutations and cyclosporine reduced Nup358 dependence. It was proposed that CypA engagement of HIV-1 capsid directs the virus through a pathway in which a subsequent Nup358Cyp-capsid interaction facilitates uncoating, nuclear entry and integration [29].

While this hypothesis has appeal as a way to connect CA-Cyclophilin protein binding with PIC transit across the nuclear pore, a number of issues need to be considered in interpreting existing data. Interaction in cells between the viral capsid and the Cyp domain of the nucleoporin itself has not been demonstrated. The capsid of the primate lentivirus SIVmac does not interact with CypA or Nup358Cyp yet SIVmac shares with all lentiviruses the property of non-dividing cell infection and it interacts with other main early event factors such as TNPO3, CPSF6 and Nup153. While the dissociation constant (Kd) for the interaction of HIV-1 CA with Nup358Cyp was initially reported on the basis of isothermal titration calorimetry (ITC) to be 16 µM (compared to 7 µM for CypA) [29], this was later revised to 94 µM [54] and Lin et al. reported an even weaker ITC-determined affinity (Kd>200 µM) [55]. HeLa have been the main cell used for Nup358 knockdowns in viral life cycle analyses [29]. These cervical cancer cells are exceptionally genetically aberrant [56] and might have been selected through their epic passage and expansion history to have distinctive nuclear import pathways. The large size of Nup358 (9.7 kb cDNA) complicates re-expression of full-length protein or specific domain-deletion mutants and these controls have not been performed. This might be critical, because knockout of murine Nup358 is embryonic lethal, as might be anticipated for a protein integral to a structure with so fundamental a role in cellular homeostasis [57], [58]. A metazoan cell translocates approximately 10^6^ cargos/second across the nuclear envelope [59]. Indeed, Hamada et al. recently showed that inactivation of the Nup358 gene in mouse embryonic fibroblasts disrupts classical NLS (cNLS)- and M9-mediated nuclear import and is rapidly lethal [58]. Importantly, preservation of nucleocytoplasmic transport and cellular viability was achieved by expression of an N-terminal Nup358 fragment harboring the leucine-rich region (LRR), three FG repeats and the first Ran-binding domain (RBD1) [58]. Corroborative evidence was provided by Wälde et al. [60]. Here we used this genetic system to assess the role of Nup358 in the lentiviral life cycle. Importantly, we were able to test in fully viable cells the specific functional relevance of Nup358 C-terminal domains, including the Cyp and SUMO E3 ligase domains. We also analyzed RNAi-mediated Nup358 depletion in human cells. For this we used not only HeLa cells but also human CD4+ T cell lines that we acutely and stably depleted of Nup358. We found that if human cells can accommodate homeostatically to loss of Nup358, and mouse cells to loss of the C-terminal half, they remain competent for viral early events.

## Results

### Derivation of knockout cell lines that express variable segments of Nup358

**Figure 1:**
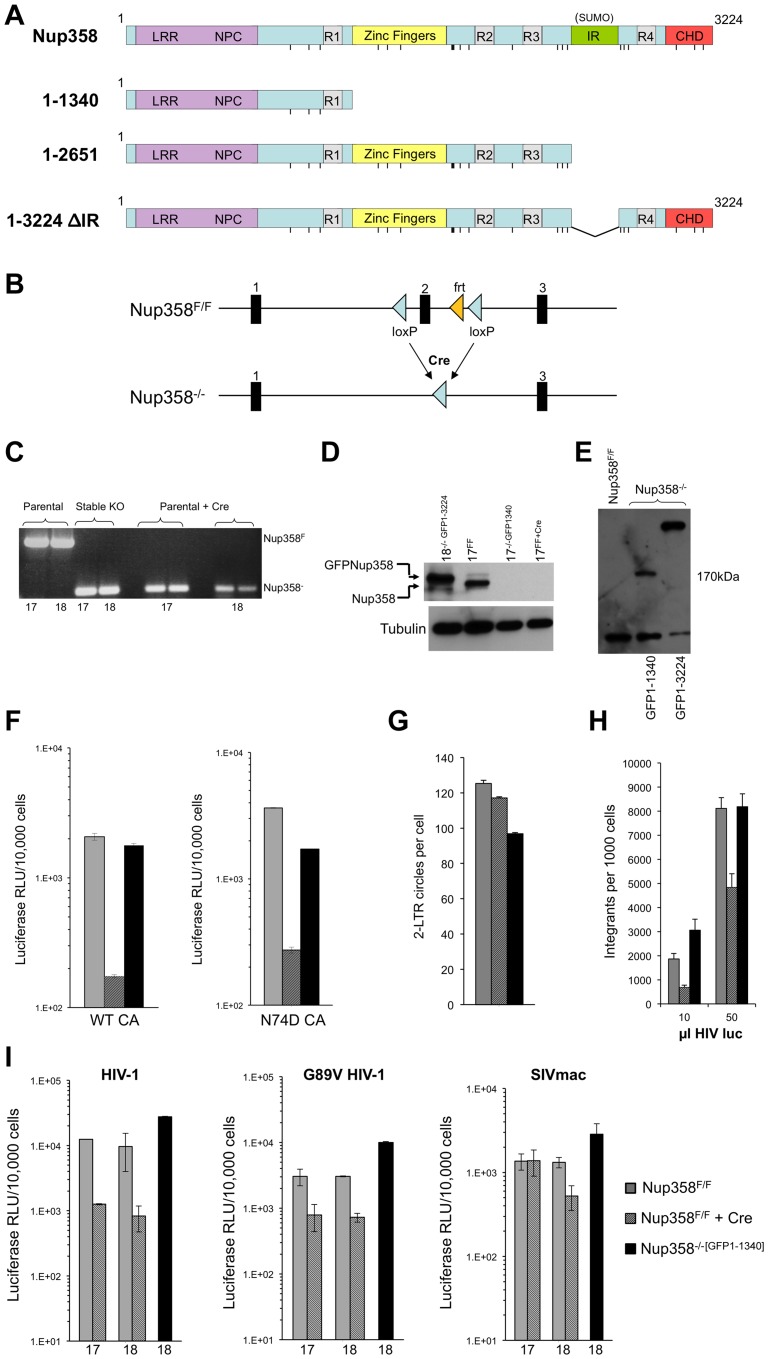
Generation of Nup358−/− cell lines. **A**) Domain structures of wild type human Nup358 and deletion mutants expressed in Nup358 null (−/−) cells. The leucine rich region (LRR) and NPC (nuclear pore complex targeting) domain mediate nuclear pore localization. The Nup358 proteins were expressed prior to Cre-lox mediated murine Nup358 gene inactivation, with eGFP fused to their N-termini. The resulting cell lines were named Nup358^−/−[GFP1-1340]^, Nup358^−/−[GFP1-2561]^, etc. Pre-complementation with GFP-1-1340 prior to Cre-mediated knockout generates a situation analogous to removing the 1,884 C-terminal amino acids of Nup358. R1-4: Ran binding domains 1–4. IR: internal repeat region, which has SUMO E3 ligase activity. CHD: cyclophilin homology domain. Vertical lines: FG repeats. **B**) Knockout cell line generation. Crossing Nup358 hypomorph mice (Nup358^H/H^) with FLPeR mice to excise an expression-attenuating *neoR* insertion yields floxed (Nup358^F/F^) mice with loxP sites in introns flanking exon 2 [58]. Thus, F/F MEFs display Nup358 expression equivalent to wild type (+/+) MEFs [58]. Three separate Nup358^F/F^ lines, numbered 17, 18 and 19, were derived from 13.5 day old embryos. Transduction of these lines with a TSIN series lentiviral vector [80] encoding Cre recombinase was used to excise exon 2, generating −/− 17, 18, and 19 cell lines that were or were not pre-complemented with the proteins shown in Figure 1A. **C**) PCR analysis of DNA isolated from parental Nup358^F/F^, stable Nup358^−/−[GFP1-1340]^ MEFs, or Nup358^F/F^ parental MEFs 6 days post Cre-expression using primers spanning exon 2. The numbers below the lanes indicate individual F/F cell lines used. Expected bands are 650 bp for an Nup358F/F locus and 120 bp for a Nup358−/− locus. **D**) Immunoblot for Nup358 in the cell lines tested in panel C with Nup358 antibody. Nup358^−/−[GFP1-3224]^ is used as a size control; note that it is slightly larger than endogenous Nup358 as predicted. Tubulin is used as a loading control. **E**) Immunoblotting of cell lines using antibody to GFP. The predicted size for GFP1-1340 is 174 Kd. These lines, derived from F/F line 18, were used in the panel F-I experiments. The lower band detected in all lanes, including GFP-lacking F/F cells, is a non-specific band detected by this antibody. **F**) Wild type reporter virus HIV-1_luc_ [80] and capsid mutant N74D HIV-1_luc_ were used to infect Nup358^F/F^, Nup358^F/F+Cre^ and Nup358^−/−[GFP1-1340]^ cells. Luciferase activity was measured 24 hours later and normalized to trypan blue-excluding cells. Error bars denote the s. d. of duplicate luciferase activity measurements in each experiment. The experiment shown is representative of N = 3 for wild type capsid and N = 2 for N74D capsid. **G**) HIV-1_luc_ D64N was used to infect the indicated cell lines and 2-LTR circles were measured in triplicate 22 hours post-infection. Each error bar denotes s. d. of 3 measurements of each sample. **H**) Integration analysis on indicated cell lines. Integration was assayed on total DNA isolated from the indicated cell lines 10 days after challenge with HIV-1_luc_ with two inputs (10 and 50 µl). Each error bar denotes s. d. of 6 measurements of each sample. **I**) Infection with HIV-1, HIV-1 G89V and SIVmac, with and without Nup358 gene deletion, and with and without GFP1-1340 pre-expression. Experiments were performed as in Fig. 1F. The *x*-axis numbers 17 and 18 refer to two independently derived MEF Nup358 knockout cell lines. For line 18, we infected in parallel cells in which, prior to Cre-mediated gene expression, we had stably expressed a protein comprised of the N-terminal 1340 amino acid segment of Nup358 (black bars). Error bars denote the s. d. of duplicate luciferase activity measurements. Experiments are representative of N = 3 for wild type capsid HIV-1, N = 2 for G89V capsid HIV-1, and N = 2 for SIVmac.

Nup358 domain structure is illustrated in Figure 1A. We derived Nup358^−/−^ mouse embryonic fibroblasts (MEFs) as previously described [58], according to the scheme shown in Figure 1B. The three independent floxed (F/F) MEF cell lines used here, designated as lines 17, 18, and 19, were derived from 13.5 day old embryos and immortalized by transduction with large T antigen. Cells were then transduced with a lentiviral vector encoding Cre recombinase, which results in exon 2 excision and simultaneously generates a frame-shift. Cre-mediated gene inactivation in Nup358^F/F^ cells causes progressive cell death starting on day 5 after transduction, with complete loss of viable cells between days 8 and 10. This correlates with loss of immunoblot-detectable Nup358 by day 6 and absent nuclear rim staining for Nup358 and RanGAP1, as well as with fundamental defects in receptor-mediated nuclear import [58]. As expression of the N-terminal 1340 amino acids preserves cNLS-mediated cargo import and cell viability [58], we expressed this and other human Nup358 deletion mutants shown in Figure 1A *prior to* Cre/lox-mediated gene inactivation, using a Tol2 transposon system [61] to express them as GFP fusion proteins (e.g., GFP1-1340).

### HIV-1 infection of Nup358^−/−^ cells

We initially compared *de novo* derived Nup358^−/−^ null cells to stable −/− cells that express the transposon-encoded N-terminal 1340 amino acids required to support sustained viability (Nup358^−/−[GFP1-1340]^ cells). Two separate Nup358^F/F^ lines (17 and 18) were transduced with a lentiviral vector encoding Cre recombinase and *pac*, with selection in puromycin commencing 24 hours later. All untransduced control line 17 and line 18 cells died in selection by 48 hours. On day 6 after Cre transduction, deletion of exon 2 was confirmed (Figure 1C) and there was no detectable Nup358 protein (Figure 1D), which coincided with the appearance of progressive cell death in the culture. Ten-fold inhibition of single cycle HIV-1 infection was observed in Nup358^−/−^ cells that remained alive (Figure 1F). Thus, Nup358 gene inactivation impairs cellular permissivity to HIV-1 infection. When just the N-terminal 1340 residue fragment was expressed prior to exon 2 excision (Nup358^−/−[GFP1-1340]^ cells, Figure 1E, lane 2), HIV-1 infectivity was rescued (Figure 1F). We also assessed N74D CA mutant virus in Nup358 null cells. This mutant arises under selection pressure in cells expressing CPSF6-358, a truncation mutant of cleavage and polyadenylation factor 6 [24]. It has been reported to render the virus independent of CPSF6-358, Nup153, Transportin-3 (TNPO3) and Nup358 [24], [25], [27], [29], [62]. N74D virus infection was equivalently inhibited in the Nup358 null state (Figure 1F), but also rescued by GFP1-1340. We did not observe significant reduction of 2-LTR circle formation in the complete absence of the protein (Figure 1G), suggesting a defect after nuclear entry. Integrated provirus formation was decreased in the Nup358-null cells and this was rescued by the GFP1-1340 fragment (Figure 1H). Therefore, the data suggest that acute loss of Nup358 impairs the ability of HIV-1 to proceed to integration after entry. Importantly, however, the cell viability-rescuing N-terminal segment containing the leucine-rich domain, three FG repeats and one Ran-binding domain – but lacking the CHD – is sufficient for rendering cells permissive to HIV-1 infection.

We also infected cells with HIV-1 capsid mutant G89V, which is defective for CypA binding. We verified that, as expected, this virus was uninhibited by owl monkey TRIMCyp (OMTC) or the same protein in which the OMTC Cyp domain was replaced by the Nup358 CHD, whereas wild type HIV-1 was potently blocked (Figure S1). These WT and G89V viruses were inhibited ten- and six-fold respectively by acute Nup358 gene inactivation but this was prevented if the 1-1340 N-terminal amino acid fragment was pre-expressed (Figure 1I). SIVmac was inhibited less by the gene excision but importantly, this difference did not depend on the CHD as, similar to HIV-1, SIVmac was unimpaired in Nup358^−/−[GFP1-1340]^ cells (Figure 1I). Therefore, relative to control cells, cells that express the N-terminal FG repeat-containing portion of Nup358 supported undiminished infection by primate lentiviruses whether or not their capsids bind cyclophilins (89G versus 89V, SIVmac) or TNPO3 (74N versus 74D).

### Infection of cells with trimmed filaments: The N-terminal 1340 amino acids of Nup358 segment support retroviral infection

**Figure 2:**
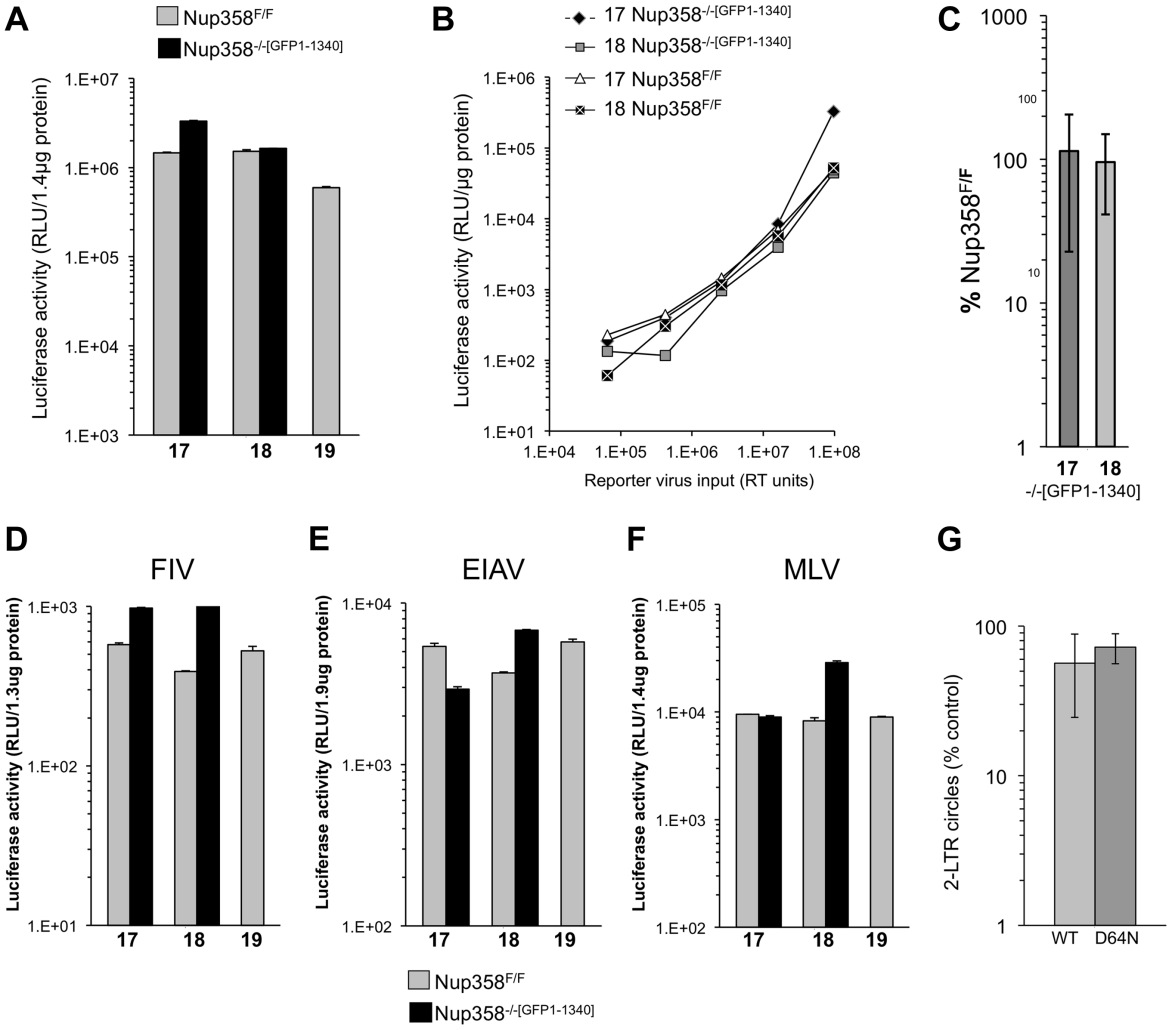
Detailed analysis of Nup358 knockout MEFs expressing the minimal Nup358 fragment, GFP1-1340. **A**) Paired cell lines (17, 18,19) either wild type Nup358^F/F^ or stable knockout Nup358^−/−[GFP1-1340]^ cells that express GFP1-1340 were infected with a VSV-G pseudotyped single cycle reporter virus HIV-1_luc_. Samples were analyzed for luciferase activity 96 hours later and values were normalized to protein concentration. Error bars indicate the standard deviation between duplicate luciferase activity measurements in each experiment. **B**) The indicated cells were infected with serial dilutions of HIV-1_luc_ reporter virus. **C**) Aggregate results of repeated experiments (n = 10) in stable Nup358^−/−[GFP1-1340]^ knockout cells, presented as a percentage of the values for parental Nup358^F/F^ cells. The mean of the ten separate infection experiments is shown. 17 and 18 refer to separate Nup358^−/−[GFP1-1340]^ stable knockout MEFs. The p-value obtained using a two tailed T test between 17 and 18 Nup358^F/F^ and Nup358^−/−[GFP1-1340]^ was 0.62 and 0.79 respectively. **D–F**) Nup358^F/F^ cells and Nup358^−/−[GFP1-1340]^ cells were transduced with luciferase-encoding retroviral vectors. (D) FIV; (E) EIAV; F) MLV. Samples were analyzed for luciferase activity 96 hours later and values were normalized to protein concentration. Error bars indicate the standard deviation between duplicate luciferase activity measurements in each experiment. **G**) Results of 2-LTR circle measurements (n = 5 experiments) in stable Nup358^−/−[GFP1-1340]^ knockout cells, presented as a percentage of the values obtained in parental Nup358^F/F^ cells are shown for an integration competent (WT) and an integration mutant (IN D64N) HIV-1_luc_. Statistical analysis was performed using two tailed T-test, p-values were 0.038 and 0.02 for WT and D64N samples respectively.

We then conducted further studies in Nup358^−/−[GFP1-1340]^ cells. We confirmed deletion of exon 2 and lack of detectable Nup358 (Figure S2). Growth curves of paired lines were similar, with less than 2 to 3 fold differences in total cell accumulation after 4 days of log phase expansion (Figure S3). Additional single cycle infections revealed no significant differences in susceptibility to infection between parental Nup358^F/F^ and cells with trimmed filaments (Figure 2A). To exclude that an excess of capsid was saturating a block in the Nup358^−/−[GFP1-1340]^ cells, we infected the same cells with serial dilutions of virus. No significant impairment in infection was apparent over a three log_10_ range (Figure 2B). The challenge experiment was then repeated ten times. The results, presented in Fig. 2C where values for GFP1-1340-repleted −/− cells are graphed as a percentage of that in F/F cells, affirmed the conclusion that infection is as efficient in cells with trimmed Nup358 filaments as in cells with intact filaments. The observed rescue is not due to over-expression of GFP1-1340, as this protein is expressed at a relatively low level (Fig. S2C).

Next we challenged the paired cell lines with additional retroviruses: feline immunodeficiency virus (FIV), equine infectious anemia virus (EIAV) and Moloney murine leukemia virus (MLV) (Figure 2D, E, F). In all cases, no significant difference was observed between Nup358^F/F^ and Nup358^−/−[GFP1-1340]^ cells. Importantly then, mouse cells lacking the C terminal 1,884 residues of Nup358, which includes the SUMO E3 ligase and cyclophilin homology domains, are normally permissive for infection with lentiviruses from each of the three main species groups (primate, feline, ungulate) as well as with a gammaretrovirus.

To assess nuclear import of viral DNA, 2-LTR circle formation was determined with an integration-competent virus and with an integrase catalytic center mutant virus (D64N). The latter was used to maximize circle formation. Aggregate data from 5 separate experiments with two independently derived MEF cell lines is presented as a percentage of 2-LTR circles seen in the parental cell lines (Figure 2G). A small but statistically significant difference in 2-LTR circle formation was seen with 1.7-fold (wild type IN, p = 0.038) and 1.3-fold (D64N, p = 0.02) less circles in Nup358^−/−[GFP1-1340]^ compared to Nup358^F/F^. No deficit in 2-LTR circle generation was apparent in knockout cells expressing full length Nup358 (Figure S4).

### Nup358 1-1340 supports HIV-1 infection in non-dividing cells

**Figure 3:**
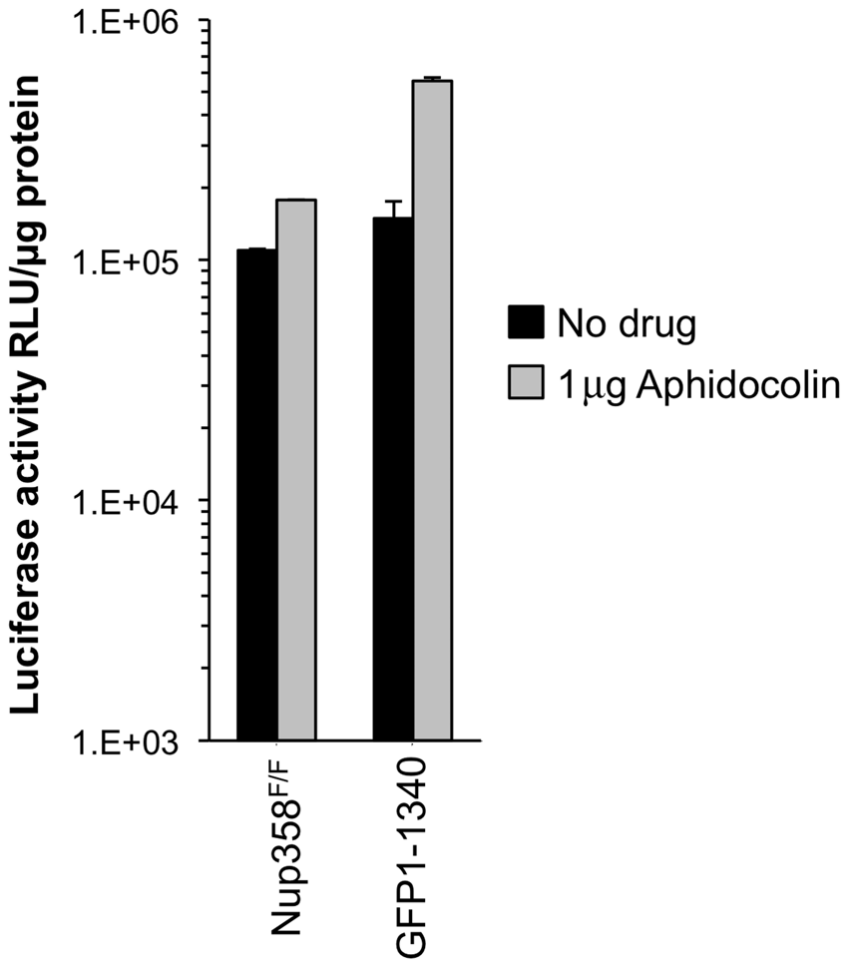
Growth arrested cell experiments. MEFs were growth-arrested in aphidicolin 1 µg/ml for 24 hours prior to infection with HIV-1_luc_. Aphidicolin was maintained throughout the experiment. Growth arrest was confirmed by FACS analysis (Figure S5). Cells were harvested for luciferase activity measurement 48 hours after infection. Error bars indicate the standard deviation for duplicate luciferase activity measurements in each experiment.

Because non-dividing cell infection by lentiviruses requires nucleopore transit, we examined the effect of cellular Nup358 status on the ability of HIV-1 to infect cells arrested in G1/S. Nup358^F/F^ and Nup358^−/−[GFP1-1340]^ cells were treated with aphidicolin for 24 hours. FACS analysis with propidium iodide showed that 98% of the cells were arrested in G1/S (Figure S5). Significant differences were not observed between cells with intact and trimmed Nup358 filaments, whether or not cells were cycling (Figure 3).

### Assessment of the SUMO E3 ligase domain and additional C-terminal segments

**Figure 4:**
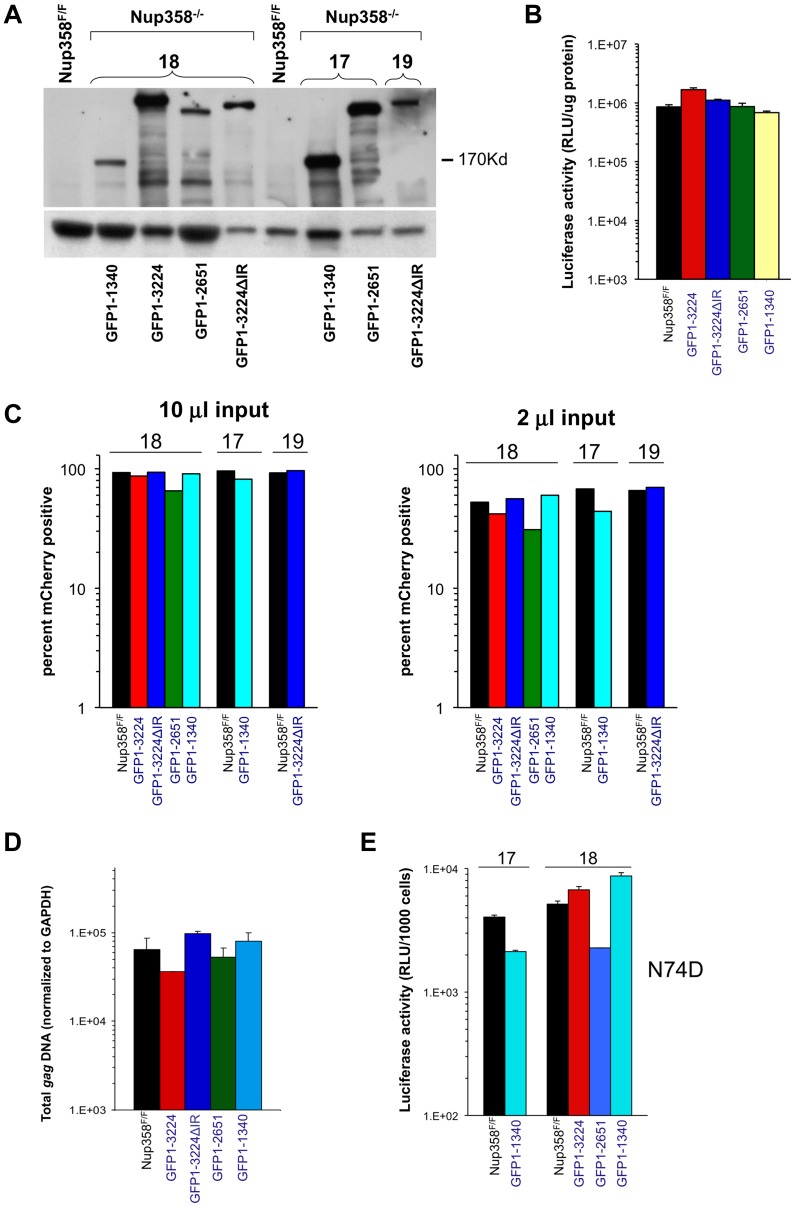
Nup358 variants with additional C-terminal regions. **A**) Immunoblot demonstrating expression of appropriate Nup358 mutant proteins. Primary antibody is to GFP. **B**) HIV-1 reporter virus challenge of indicated cell lines. **C**) HIV-1 mCherry vector challenge of indicated cells. Cells were transduced with vector TsinCherry and analyzed by FACs 72 hrs later. Two separate infections at two inputs (10 µl and 2 µl) are shown. 10,000 events were collected per sample. **D**) Total integrated *gag* DNA copies in the indicated MEF cells were measured ten days post transduction. Results are normalized to GAPDH copies. **E**) Indicated cell lines were challenged with an HIV-1_luc_ N74D capsid mutant virus. Luciferase activity was analyzed as described. Error bars indicate the standard deviation between duplicate luciferase activity measurements in each experiment. 17 and 18 refer to independently derived MEF cell lines. This experiment was repeated twice with similar results.

Additional Nup358 −/− cell lines that express human Nup358 variants containing more C-terminal regions were analyzed next. GFP1-2561 contains zinc finger domains and two additional Ran-binding domains but lacks the Cyp domain, while GFP1-3224ΔIR lacks only the SUMO E3 ligase domain (Figure 1A). GFP1-3224 is a fusion of eGFP to all of Nup358. As the Nup358 allele used for all of our experiments is the human one, GFP1-3224ΔIR and GFP1-3224 allowed us to also ask whether the human Nup358Cyp domain has unique properties versus the murine domain. (Figure S6 shows an alignment of the mouse and human Nup358Cyp domains). Deletion of exon 2 was verified (Figure S7) and expression of GFP-Nup358 mutant proteins of predicted size was confirmed (Figure 4A). HIV-1_luc_ infection challenge did not reveal significant differences in susceptibility to infection in three of the four cell lines compared to F/F cells (Figure 4B). We did consistently observe moderately higher luciferase activity levels in HIV-1_luc_-infected MEFs expressing full length human Nup358 (GFP1-3224 cells, Figure 4B and Figure S8A). The reason for this is not clear, but immunoblotting also indicated that there is more full length human protein expressed in these cells than full length murine protein in F/F cells (see Figure S8B). Challenges with an HIV-1 vector in which expression is under the control of an internal CMV promoter corroborated these results, but in this case, no discrepancy between GFP1-3224 cells and the other lines was observed (Figure 4C). Total integrated *gag* DNA copies were similar in all the cell lines as well (Figure 4D). Lastly, we assessed N74D CA mutant virus. As Figure 4E shows, the mutant virus behaved similarly to wild type HIV-1 in the presence and absence of full length Nup358, suggesting that whether or not HIV-1 utilizes the alternative nuclear import pathway that has been hypothesized for the N74D mutant virus and FIV [24], the presence of the Cyp and SUMO domains in Nup358 is not highly consequential.

### HIV-1 requirement for TNPO3 in Nup358^FF^ and Nup358^−/−[GFP1-1340]^ MEFs

**Figure 5:**
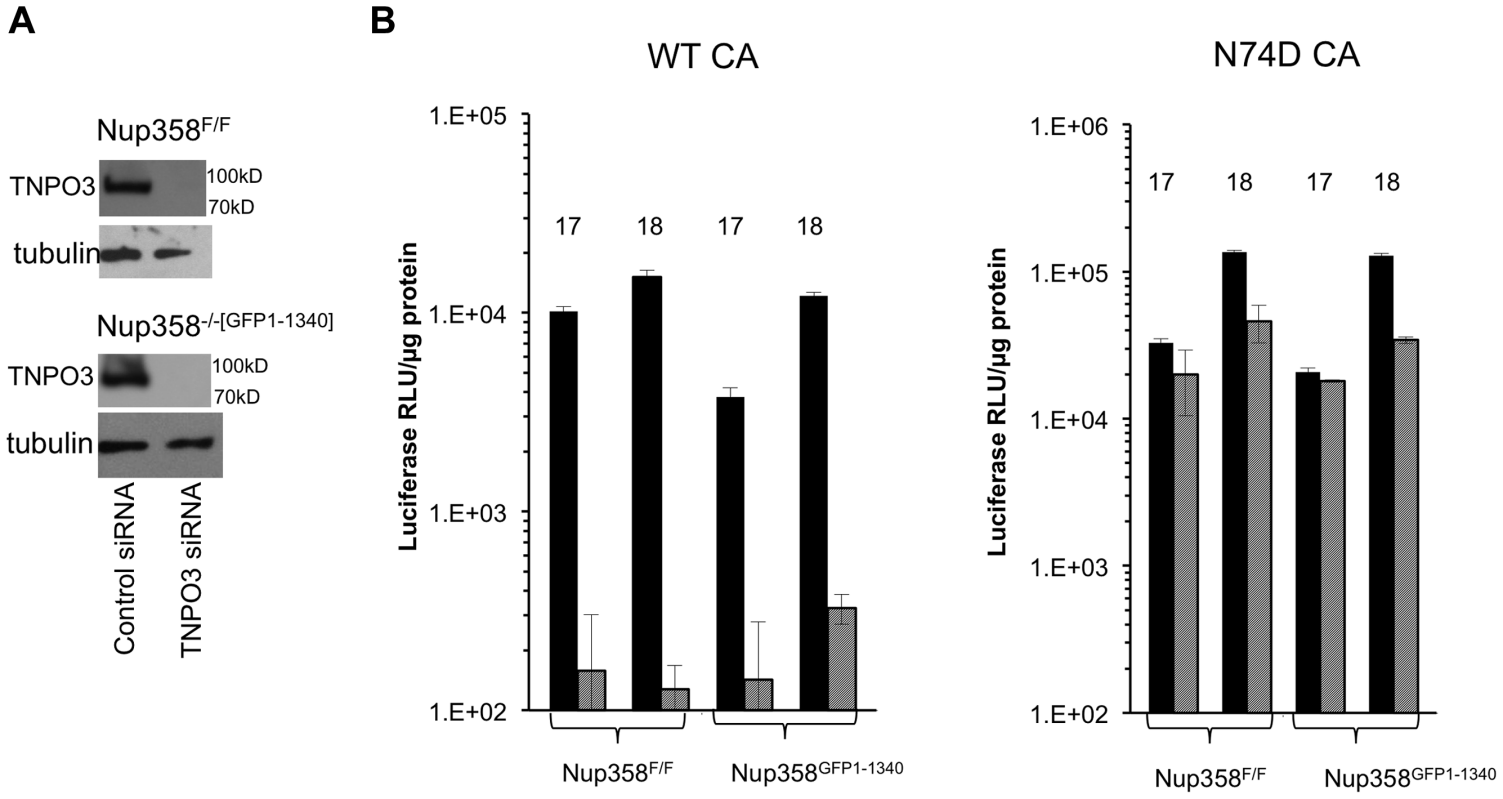
HIV-1 TNPO3 dependence is the same in human and mouse cells. **A**) Indicated cell lines were transfected with control siRNA or siRNA for TNP03. At 72 hours after transfection, cells were harvested and analyzed for TNPO3 protein levels. Tubulin loading control is shown also. **B**) MEFs treated with siRNAs (solid black bars: control siRNA; hatched gray bars: TNPO3 siRNA) were challenged with WT or N74D HIV-1 reporter virus. Intracellular luciferase activities were determined 72 hrs after infection. 17 and 18 refer to two independently derived MEF Nup358 knockout cell lines. P value, obtained using a two tailed T test, for the difference between control compared to TNPO3 siRNA is 0.005.

To assess the similarity of mouse and human early event dependency factor systems, we depleted the karyopherin β TNPO3 (Transportin-3) in Nup358^FF^ and Nup358^−/−[GFP1-1340]^ cells (Figure 5A). TNPO3 has been implicated in HIV-1 early events, with conflicting evidence for nuclear import and post-import roles and for integrase or capsid as the principal viral interactor with this protein [19], [22], [23], [27], [28], [62]–[66]; Indirect action via effects on intracellular CPSF6 location may also be important [67], [68]. Immunoblotting at 72 hours after siRNA addition confirmed TNPO3 depletion (Figure 5A). The cell lines were challenged with WT and N74D reporter viruses. Figure 5B shows that the WT virus is strongly dependent on TNPO3 in Nup358^F/F^ and Nup358^−/−[GFP1-1340]^ cells but the N74D virus is not. This strong phenotype contrasts with the results obtained with Nup358 depletion in our study. They are consistent with previously published results in human cells and they suggest that HIV-1 utilizes common nuclear import pathways in the cells of both mammals. The results establish as well that TNPO3 dependency does not depend upon the C-terminal 1,884 amino acids of Nup358.

### Functional equivalence of the mouse and human versions of CypA and Nup358Cyp

**Figure 6:**
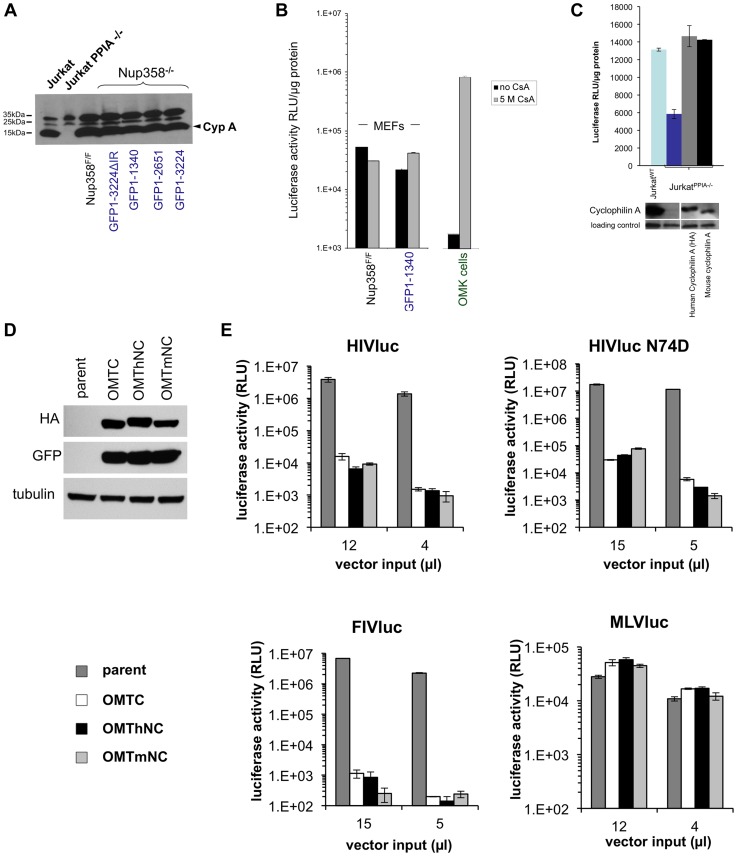
Human and mouse cyclophilin domains are functionally similar. **A**) CypA protein levels in Nup358^F/F^ and Nup358^−/−[GFP1-1340]^ cell lines. Wild type and knockout Jurkat cells were included as positive and negative controls respectively. **B**) Cyclosporine (Cs) has minimal effect on HIV infection in the presence or absence of the C-terminal domain. Indicated cell lines were transduced with HIV-1 reporter virus in the presence of absence of 5 µM Cs. OMK cells were included in the experiment as a positive control. **C**) Jurkat PPIA−/− cells were transduced with lentiviral vectors expressing either a HA tagged human or untagged mouse cyclophilin. Cells expressing the indicated constructs were challenged with HIV reporter virus (top panel). Cyclophilin expression was confirmed using antibody to CypA (lower panel). **D**) TRIM-CHD fusion proteins. The Cyp domain of owl monkey TRIMCyp (OMTC) was replaced with the human or mouse Nup358 CHD (creating proteins OMThNC and OMTmNC respectively). The proteins were expressed stably in CrFK cells, which lack endogenous TRIM5alpha or TRIMCyp restriction. Cells were transduced with dual promoter vectors that express GFP and OMTC, OMThNC or OMTmNC. Expression of appropriate sized fusion proteins was confirmed by western blotting with rat anti-HA. Equivalent GFP expression was also observed. Tubulin was immunoblotted as a loading control. **E**) Parental and restriction factor-expressing CrFK cells (OMTC, OMThNC, OMTmNC) were challenged with WT HIV-1_luc_, N74D HIV-1_luc_,, FIV_luc_, and NB-MLV_luc_. Luciferase activity was measured at 2 days post transduction and normalized to cell number.

The role of the cellular prolyl isomerase CypA in lentiviral life cycles is complex [69]. HIV-1 capsid interaction with CypA facilitates HIV-1 infection in most human cells. In rhesus macaque or African green monkey cells, interaction with CypA paradoxically decreases HIV-1 infectivity by facilitating TRIM5alpha-mediated restriction. On the other hand, CypA does not interact with the primate lentivirus SIVmac. Its role in lentiviral life cycles has been investigated for two decades but remains incompletely understood [69]. Schaller et al. recently proposed that engagement of cytoplasmic CypA by capsid is the first step of a nuclear transport pathway involving TNPO3, Nup358, and Nup153 [29]. They reported that, unlike the HIV-1 CA-CypA interaction, *in vitro* binding of assembled capsids to the Nup358 Cyp domain is cyclosporine-insensitive. Intracellular restriction of HIV-1 by TRIM-Nup358Cyp was, unlike TRIMCyp restriction, also not rescued by cyclosporine, but this drug did rescue the virus from cellular Nup358 depletion [29]. Two groups subsequently presented evidence for a model in which CypA shields incoming HIV-1 from detection by a cytosolic DNA sensor [45], [46]. CypA is highly conserved in mammals and the amino acid sequences of mouse and human CypA are 98% identical. Here, we first ascertained that levels of CypA were comparable between Nup358^F/F^ and Nup358^−/−[GFP1-1340]^ cells (Figure 6A). We then tested whether cyclosporine impacted viral phenotypes in a Nup358-dependent manner. When 5 µM cyclosporine was added to cells at the point of infection, no difference was noted (Figure 6B). This was in clear contrast to owl monkey kidney cells (positive control), where this induced a marked rescue from TRIMCyp inhibition as expected [53].

We next expressed mouse CypA in CypA gene knockout (PPIA^−/−^) human Jurkat cells [44] to determine if it can substitute functionally for human CypA. Mouse and human CypA each rescued the HIV infectivity defect in PPIA^−/−^ cells, showing functional equivalence of the two CypA proteins (Figure 6C). Finally, as noted above, TRIMNup358Cyp inhibits both WT and N74D HIV-1 [29]. We tested if mouse Nup358Cyp and human Nup358Cyp were similarly antiviral when used in this way to replace the Cyp domain of owl monkey TRIMCyp (Figure 6D,E). Equivalent expression of the TRIMhNup358Cyp and proteins was confirmed (Figure 6D) and the effects on HIV-1 WT, HIV-1 N74D, FIV and MLV were determined (Figure 6E). The lentiviruses, but not MLV, were inhibited equally by both OMThNC and OMTmNC (Figure 6E). Therefore mouse and human Nup358Cyp can bind lentiviral capsids equivalently and this binding is dependent on the CA cyclophilin binding domain.

### Nup358-depleted human CD4+ T cells

**Figure 7:**
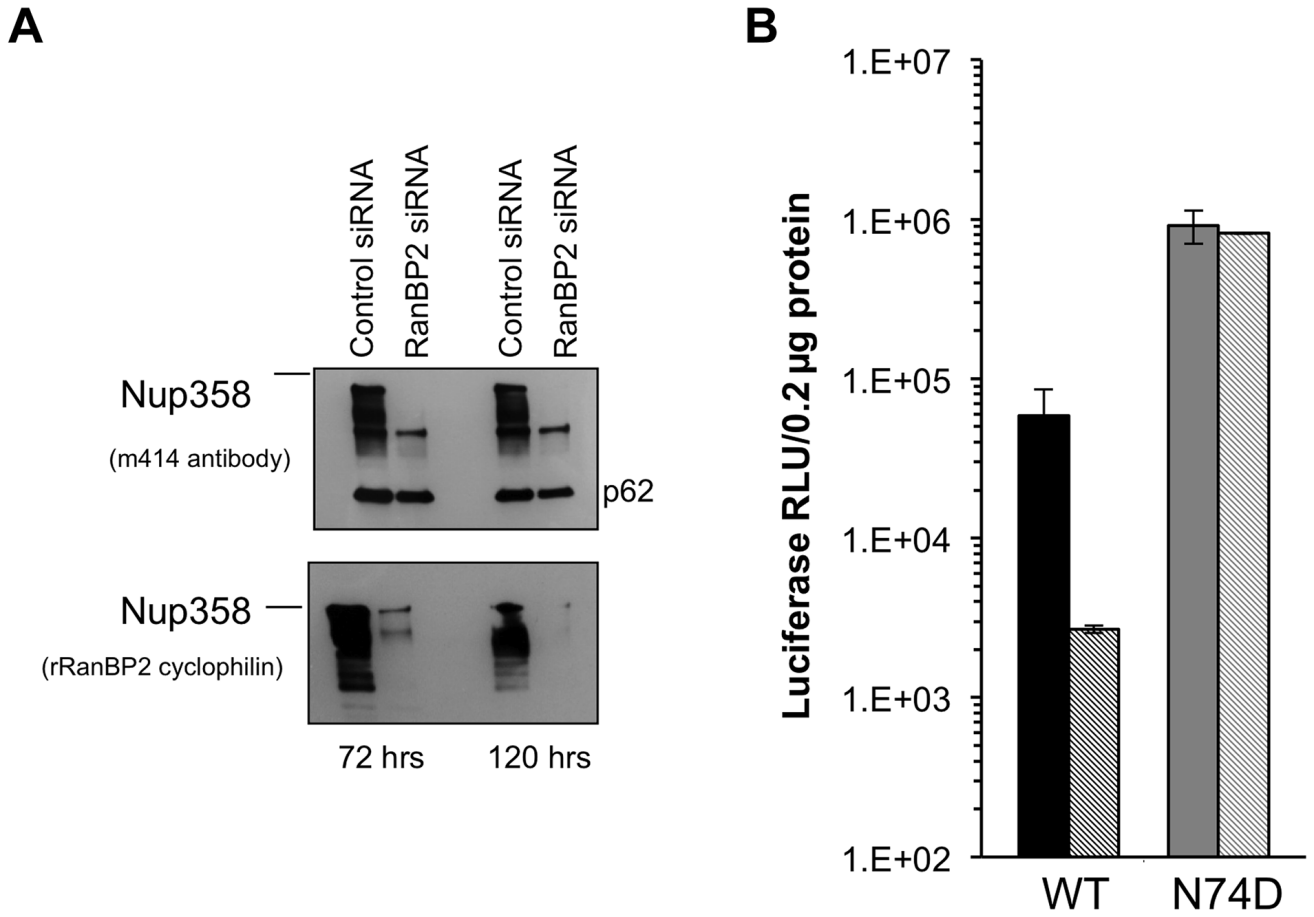
Acute depletion of Nup358 in Hela cells. **A**) Hela cells were transfected with control siRNA or Nup358 specific siRNA. Cells were harvested at 72 and 120 hours and analysed by western blot with two different antibodies. The m414 antibody detects several FG containing nucleoporins, such that the lower molecular weight nucleoporins serve as loading controls and evidence for specific Nup358 depletion. **B**) At 72 hours, the transfected cells were challenged with WT or N74D CA HIV-1_luc_ and analysed 48 hours after for luciferase activities.

**Figure 8:**
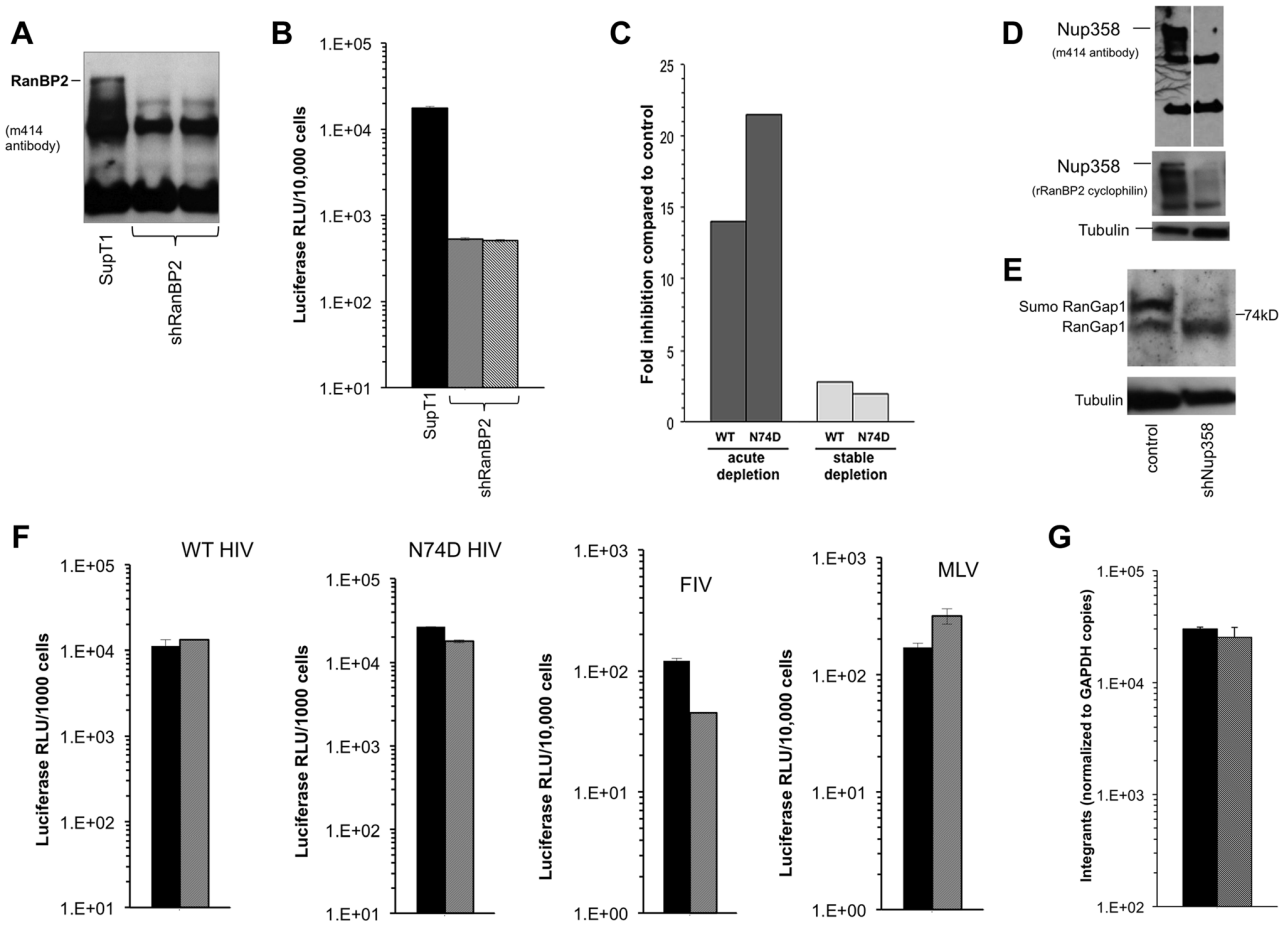
Acute and stable Nup358 depletion in human CD4+ T cells. **A**) SupT1 cells were transduced with a lentiviral vector that co-expresses mCherry and a Nup358-targeted shRNA. Four days after transduction, cells were uniformly mCherry-positive and protein depletion was confirmed using mab414. Two separate cell lines were generated (second and third lanes of immunoblot). **B**) At the same time that cells were sampled for the immunoblotting shown in (A), they were challenged with HIV-1_luc_ and luciferase activities were analyzed at 48 hours after infection. **C**) Infection of Supt1 cells with HIV-1 and HIV-1 N74D capsid mutant reporter viruses after acute (left) and stable (right) knockdown. See panel 8D and associated text for description of the stable line. **D**) SupT1 cells 6 weeks after shRNA transduction with two different antibodies confirms persistent knockdown of Nup358. The two lanes in the top immunoblot are from the same film of the same gel. Note that the smaller nucleoporins detected by mAb414 confirm equal loading. **E**) Equal numbers of cells from parental SupT1 or stable shRNA knockdown cells were lysed and used in western blot for RanGAP1. In the absence of Nup358, sumoylated RanGAP-1 levels decrease, and there is an increase in un sumoylated parent. **F**) Control (black bars) and stable Nup358 knockdown cells (hatched bars) were challenged with HIV-1_luc_ (WT or N74D), FIV_luc_, and MLV_luc_. **G**) Integration levels in control (black bar) and stably Nup358-depleted SupT1 cells (hatched bars) were analyzed by Alu-PCR.

We inferred from the foregoing results that there is substantial conservation of HIV-1 nuclear import pathways between mouse and human cells. The effects of acute Nup358 depletion could be indirect and depend on the important role this protein has in maintaining nucleocytoplasmic transport of many cellular cargos and transport receptors and hence in preserving homeostasis. Alternatively, the N-terminal segment containing the three FG repeats may interact directly with the PIC component or a PIC-associated transport receptor. Previously reported Nup358 knockdown experiments have used HeLa cells frequently [24], [26], [29]. Indeed we confirmed the inhibitory effect of Nup358 knockdown in these cells, as well as resistance of the N74D capsid-mutant virus (Figure 7A,B). However, because HeLa cells are not representative of the normal in vivo CD4+ T cell targets of HIV-1 and have also accumulated an extreme burden of genetic aberrations ([56]; see also [70]), they might be unrepresentative in pertinent respects, especially nuclear transport pathways. We therefore carried out transient and stable Nup358 depletions in an HIV-1-susceptible human CD4+ T lymphocyte cell line, SupT1 (Figure 8).

Single cycle HIV-1 infection was inhibited when SupT1 cells were depleted acutely with Nup358 shRNA-transducing lentiviral vectors (Figure 8A–C). Infections with normalized inputs showed that CypA binding mutant HIV-1 (G89A) had reduced infectivity compared to wild type HIV-1 in the parental and knocked down cells (Figure S9). The HIV-1 N74D CA mutant was also inhibited by the Nup358 depletion (Fig. 8C, left columns). Similar to what we observed when Nup358 was abruptly depleted in primary mouse cells by Cre excision of the gene, there was temporary growth cessation followed by visible cell death in the culture 7–10 days after knockdown. However subsequent passage of the shRNA-transduced SupT1 cells yielded stably Nup358-depleted cells that were indistinguishable from the parental cell line in growth rate, maximum culture density, individual cell morphology and cluster morphology. Nup358 remained depleted (Figure 8D). SUMO-1 conjugated RanGap1 requires Nup358 for protection from SUMO-1 isopeptidases [58]. Confirming functionally the immunoblotting with the two antibodies, there was little detectable sumoylated RanGap1 (77 kDa band) and a concomitant increase in unconjugated RanGap1 (65 kD band) in the stable Nup358 depleted cells (Figure 8E). However, in these healthy Nup358-deficient human CD4+ T cells there was no significant inhibition of HIV-1, N74D HIV-1, FIV or MLV (Figure 8C,F). Integration levels in control and Nup358-depleted cells were identical (Figure 8G).

**Figure 9:**
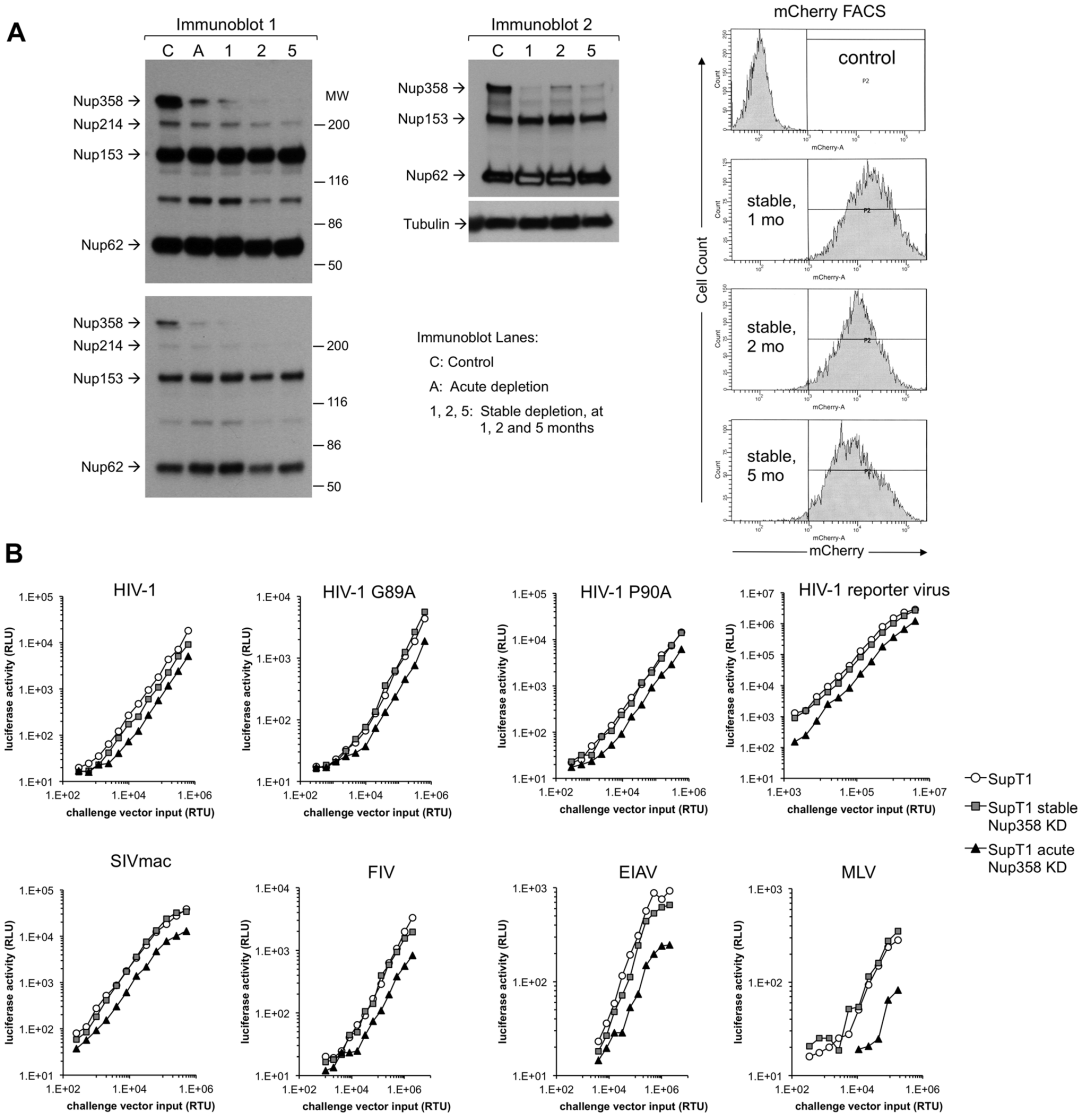
Verification of SupT1 knockdown in acutely and stably depleted cells and challenge with HIV-1, HIV-1 G89A, HIV-1 P90A, SIVmac, FIV, EIAV and MLV. **A**) Extent, specificity and stability of shRNA knockdown. Cells frozen 1, 2, and 5 months after stable depletion by shRNA transduction were thawed, passaged for ten days and lysates were immunoblotted with mAb 414, which recognizes Nup358, Nup214, Nup153 and Nup62. Two different exposures of the left immunoblot are shown. A second immunoblot of the stable cell lines was also done (right blot). The mCherry marker co-encoded by the shRNA-transducing lentiviral vector was assessed by FACS to further verify stability of expression. These experiments were done simultaneously with the viral challenges shown in Fig. 9B. MW: molecular weight. C: control. A: acute. 1, 2, 5: cells assessed at 1, 2, and 5 months after stable cell line derivation. **B**) Control cells, acutely depleted cells and stably depleted cells that were assessed in panel A by immunoblotting (lanes C, A and 1 respectively) and FACS were challenged with HIV-1, HIV-1 G89A, HIV-1 P90A, SIVmac, FIV, EIAV and MLV vectors. G89V vector gave the same result as G89A vector (data not shown).

To assess viral dependence further, SupT1 cell lines were assessed for extent, specificity and stability of Nup358 depletion (Figure 9A) and simultaneously infected with multiple different retroviruses (Figure 9B). The RNAi was highly specific to Nup358 as shown by probing with an antibody that detects it and three other FG repeat nucleoporins (Figure 9A). In addition, the knockdown was stable, persisting over 5 months in culture. SIVmac showed the same profile as HIV-1 in single input experiments (Figure S10). We challenged cells over a range of viral inputs with this virus, HIV-1, HIV-1 cyclophilin binding mutant capsid viruses (P90A, G89A, G89V), as well as FIV, EIAV and MLV (Figure 9B). MLV was the most affected by acute depletion, consistent with the growth-arresting effect, with approximately a one log shift to the right. For the lentiviruses, there was moderate infection impairment after acute Nup358 depletion whether the virus capsid protein can (HIV-1) or cannot (SIVmac, HIV-1 P90A, G89A, G89V) bind the Nup358 CHD. With the exception of a slight inhibitory effect on EIAV, the tested viruses were unimpaired in stably depleted SupT1 cells. Thus, in this human CD4+ T cell line, capsid ability to bind cyclophilins did not correlate with viral phenotypic response to acute or stable Nup358 depletions.

## Discussion

We have examined the role of Nup358/RanBP2 in the HIV-1 life cycle using a conditional knockout system that completely eradicates the protein from cells, as well as human CD4+ T cells depleted with RNAi. Our results are in agreement with previous knockdown studies in concluding that acute depletion of Nup358 does clearly inhibit HIV-1 infection [19], [20], [24], [26], [28], [29]. This is the first study to carry out domain analyses and back-complementation studies and the first to use human CD4+ T cells. We emphasize four main observations. One is that the N-terminal 1340 amino acids of Nup358 are sufficient to prevent loss of viability and major nuclear import pathway competence in Nup358 gene-knockout cells [58], [60]. A critical housekeeping function of Nup358 appears to be the capture of recycling RanGTP–importin-β complexes at the cytoplasmic face of the nuclear pore, thus maintaining the Ran-GTP cycle, cNLS-mediated cargo import and cellular health. The 1340 amino acid segment with RBD1 is sufficient, while deletion of RBD1 abrogates cNLS-containing cargo import [58]. Acute removal of Nup358 may alter the nucleocytoplasmic transport of many cellular cargos. Our experiments establish that the N-terminal portion with one Ran-binding domain and three FG repeats is sufficient for maintaining lentiviral competence to proceed after entry to integration and proviral transcription. Thus, our results indicate that Nup358 does play an important role in the HIV-1 life cycle, albeit one that is more likely to depend on the FG repeats, akin to the role recently demonstrated for these elements in Nup153 [71], rather than the CHD.

A second important observation was made in immortalized human CD4+ T cells. Here similar acute toxicity and concomitant decreases in HIV-1 infection were observed after RNAi-mediated depletion. However, when these cells adapted to stable Nup358 deficiency, HIV-1 infection was not impaired (Figure 8F, Figure 9). In both mouse cells and SupT1 cells, if the global cellular alterations caused by abrupt Nup358 loss are ameliorated, viral infection is concomitantly rescued. The toxicity observed in both abruptly gene-inactivated mouse cells and in abruptly knocked down SupT1 cells shows that significantly altered cellular physiology can follow sudden deprivation of Nup358. Comparable inhibition of lentiviruses that do or do not bind cyclophilins in acutely depleted mouse (Figure 1I) and human cells (Figure 9) suggests a CHD-independent virological role. That HIV-1 is not blocked in stably Nup358-depleted SupT1 cells supports a cellular homeostasis disruption model rather than a cofactor role mediated through the Nup358 CHD.

Third, we obtained evidence that viral nuclear import pathway(s) are substantially conserved between mouse and human cells. We show that TNPO3 facilitates infection in MEFs as in human cells. This is independent of the Nup358Cyp domain and was apparent in both WT MEFs and cells where only the Nup358 N-terminal amino acids were present. Fusions of mouse Nup358Cyp or human Nup358Cyp to owl monkey TRIM equivalently inhibited both WT and N74D HIV-1. Therefore, both species Nup358Cyp domains function similarly in this assay. We also show that mouse CypA can rescue the CypA deficit in Jurkat PPIA^−/−^ cells. Thus the murine elements of the proposed sequential cyclophilin binding pathway are functional.

Fourth, we analyzed the phenotypes of HIV-1 capsid mutants, including the TNPO3 interaction-defective N74D, and cyclophilin binding mutants P90A, G89A, and G89V. We further tested SIVmac, the capsid of which does not bind CypA or the Nup358 CHD. These experiments did not identify Nup358Cyp dependence in mouse or human cells. The roles of CypA-capsid interactions in lentiviral replication are complex and they remain incompletely understood. In HeLa cells and monocyte-derived macrophages (MDM), differential effects between wild type HIV-1 and such viruses led to the proposal that a sequential CypA-Nup358 CHD interaction regulates core uncoating and that the failure of P90A HIV-1 to propagate in MDM (and the block imposed in these cells by CsA) may be due to inability to access this pathway [29]. However, more recent studies in MDMs and dendritic cells attribute this block to a different model in which CypA binding to CA acts to shield against activation of the cytosolic DNA sensor cGAS [45], [46]. It is likely that the perturbations that follow sudden depletion of a key nucleoporin can differentially impact the infectivities of viruses that do or do not bind CypA through mechanisms that do not depend upon the Nup358 CHD. Here, in mouse cells, SIVmac was less affected than HIV-1 by acute Nup358 depletion but both were unaffected when the FG repeat domain-containing, CHD-minus N-terminal 1340 amino acids were present (Figure 1I). In SupT1 cells, on the other hand, acute Nup358 depletion diminished the infectivity of viruses that can or cannot bind CypA similarly (Figure 9).

There are now three instances in which the HIV-1 life cycle has been evaluated in stable cell lines in which Nup358 has been knocked out or depleted: the Nup358^−/−[GFP1-1340]^ MEFs and SupT1 cells (present work), and the HeLa cell clones of Schaller et al. The data taken together may suggest that HeLa cells and SupT1 cells accommodate differently to persist in the stable Nup358-depleted state, with SupT1 cells adapting in such a way that HIV-1 nuclear import is not impaired. Cell, tissue and context dependent differences in nuclear pore composition may impact how a given cell type tolerates nucleoporin depletion [72]. The Nup358 −/− MEFs studied here are very informative in this regard as they differ from all the present and prior Nup358 knockdowns in completely lacking any Nup358 protein. These cells are unable to adapt and survive without pre-repletion of the 1–1340 N-terminal segment that contains RBD1 and three FG repeats, but then are able to support undiminished HIV-1 infection. The dispensability of the CHD, and conversely the importance of the N-terminal FG repeat-containing segment, for HIV nuclear import in these cells is a departure from the proposed sequential cyclophilin-binding model. However, given this protein's central role in nuclear import, it is not a surprising one. RBD1 is essential for recycling importin-β to the nuclear rim. A number of karyopherin-β family members, such as importin-β, importin-7 and TNPO3 have been implicated in PIC nuclear import [21], [73], [74]. TNPO3 been suggested to facilitate nuclear import and integration, with both integrase and capsid implicated in various studies as the viral determinant [19], [22], [23], [27], [28], [62]–[67]. Because Nup358 is important for nuclear shuttling of transportin and importin-β [58], [75], it may also be involved in TNPO3 activity. In HeLa cells, knockdown of Nup358 led to TNPO3 relocalization away from the nuclear rim and into the cytoplasm [76]. Thus proper trafficking of TNPO3 could be Nup358-dependent. Nup358 has also been shown to enter a cytoplasmic pool that colocalizes with interphase microtubules [51]. The N-terminal 1–900 amino acids are required for this association. This is another possible route whereby Nup358 might support viral nuclear import. It remains possible also that Nup358 serves more than one role in the lentiviral life cycle and this may be cell type-specific. A recent study found that binding to in vitro assembled HIV CA-NC complexes was similar for Nup358 proteins having or lacking the cyclophilin homology domain, suggesting that additional regions of Nup358 may play a role in CA binding [77].

Acutely gene-disrupted mouse cells display impaired infection by WT and N74D HIV-1. While the N74D virus replicates poorly in macrophages, and this has been interpreted as consistent with use of a Nup358-independent nuclear import pathway [29], a recent study suggested that this block in macrophages is instead due to an earlier block, prior to reverse transcription [78]. Additionally, the N74D virus is actually hypersensitive to cyclosporine [78] despite its apparent diversion from the hypothesized CypA-Nup358Cyp pathway, and it at the same time remains sensitive to TRIM-Nup358Cyp inhibition ([29], and the present study). Its resistance to TNPO3 depletion, which we here confirm (Figure 5 and 7), indicates that numerous factors may determine the phenotype of this virus.

Unlike the −/− MEFs, viable Nup358-depleted SupT1 cells could be derived without re-expression of any part of the protein. Thus, these human CD4+ T cells are able to adapt to the lack of Nup358 and remain fully permissive to HIV-1. The notion that HIV-1 can utilize nuclear import mechanisms flexibly [24], with which our study is consistent, is also likely to apply to diverse cellular cargos. It will be of interest to determine the reasons underlying the different adaptability of these mouse fibroblasts and human CD4+ T cells to Nup358 deprivation, as this may shed light on hypothesized alternate nuclear import pathways. It will also be of interest to determine HIV-1 integration site distribution patterns in our cell lines, as it remains possible that although overall levels of integration and early gene expression were not substantially affected, integration targeting may differ.

In summary, permissivity to HIV-1 early events is preserved if cells achieve cellular homeostasis after Nup358 loss (loss of the whole protein in the case of the human T cells and effective loss of the C-terminal 1,884 amino acids in the conditional knockout mouse fibroblasts). Viral phenotypes may reflect two main and not mutually exclusive sequelae to removal of a major nucleoporin from the NPC: secondary effects resulting from systems biology-level perturbations as the nucleocytoplasmic trafficking of ca. one million cellular cargos per second is altered, or as suggested by the gene knockout cells pre-complemented with the N-terminal 1340 amino acids, there may be a direct cofactor role in which binding of Nup358 to a viral component or PIC-bound transport receptor is functionally important. Similar to Nup153 [71], this may involve engagement by FG repeats, which are present in that N-terminal region as well as in most other portions of the protein, even in the cyclophilin domain (Fig. 1A).

## Materials and Methods

### Nup358^−/−^ cells

Animal research was performed with the written approval of the Mayo Clinic Institutional Animal Care and Use Committee in accordance to all federal, state, and local guidelines and with fidelity to the guidelines in The Guide for the Care and Use of Laboratory Animals of the National Institutes of Health and the accreditation and guidelines of the Association for the Assessment and Accreditation of Laboratory Animal Care. Nup358^−/−^ mouse embryonic fibroblasts were derived as described [58]. Nup358^H/H^ (hypomorph) mice were crossed with FLPeR transgenic mice. Three independent MEF cell lines were derived from 13.5 day old embryos. These were immortalized by transduction with murine stem cell retrovirus expressing large T antigen. Cells were then transduced with a lentiviral vector encoding Cre recombinase to remove exon 2, which frameshifts Nup358. Re-expression of human RanPB2 deletion mutants was achieved using a Tol2 transposon system [61]. As previously observed by Hamada et al. (ref [58]; see Fig. S5 therein), −/− MEFs pre-complemented with GFP1-1340 express relatively low levels of this protein. Fig. S2 shows flow cytometry. They are not detectably GFP-fluorescent by standard epifluorescence microscopy. MEFs were maintained in DMEM with 10% FBC, PSG, Na pyruvate, Non essential amino acids and Beta mercaptoethanol. To generate Nup358 null cells, 400,000^F/F^ cells were plated per well of a 6 well. These were transduced with TsinCrePuro. 24 hours post transduction the cells were placed in puromycin 3 ug/ml selection. Control cells died after 48 hours. On day 6, cells were washed and a fraction removed for PCR and western blotting (see below). Total DNA was obtained using a DNeasy kit (Invitrogen) and PCR across exon 2 performed as described. Cells were then plated for HIV-1 challenge. 100,000 or 200,000 trypan blue excluding cells were plated per well of a 24 well and infected 6 hours later. 24 hours after challenge trypan blue excluding cells were counted and luciferase activity was assayed according to the manufacturers protocol. Assay of HIV integrants in murine cells was performed as described, using the BBL-1 PCR assay [79].

### Immunoblotting

Western blot for full length mouse Nup358 was performed as described [58] using a rabbit antibody directed at the CypA epitope, or the mab414 from Covance which detects FG repeat containing nucleoporins. GFP fusion proteins were detected using Living Colors *Aequorea victoria* green fluorescent protein monoclonal antibody JL-8. Mouse anti-α-tubulin mAb (clone B-5-1-2; Sigma) was used as a loading control of whole cell lysates. TNPO3 antibody: Epitomics cat no. 3824-1. RanGap1 antibody: N19 Santa Cruz.

### RNAi

For Nup358 siRNAs, 30,000 Hela cells were plated per well of 24 well the day before transfection. Control siRNA (Dharmacon D-001810-01-05, ON-TARGETplus Non-targeting siRNA #1) or Nup358 siRNA(Dharmacon J-004746-09-0005, ON-TARGETplus siRNA, Human RANBP2 (5903)) were resuspended in 1X siRNA buffer as per manufacturers recommendations. Cells were transfected overnight with 50 nM of each siRNA using Dharmafect 1 in Accell serum free media. siRNA mix was removed next day and regular media added. Cells were harvested for western blots using antibodies indicated above and HIV infection as noted 72 hours after transfection. For Nup358 shRNAs, a lentiviral vector co-encoding mCherry and an shRNA was used [80]. The shRNA targets Nup358 sequence GCGAAGTGATGATATGTTT was used. The same shRNA was used by Schaller et al. in HeLa cells [29]. Vector was produced at large scale in Cell Factories (Nunc, Nalperville, IL) as described [81], [82] and banked in single aliquots for repeated use. Knockdown correlated with mCherry-positivity and equivalently fluorescent cells were taken into viral challenge experiments. SupT1 cells were transduced with the vector and protein expression analyzed at different time points. Cells were challenged with lentiviral reporter viruses as described [83].

### TNPO3

25,000 MEFs were plated per well of 24 well. Cells were treated with 1 µM Accell siRNA control (Dharmacon D-001910-01-05, Accell Non-targeting siRNA #1) or an Accell mouse TNPO3 smartpool (Dharmacon E-066710-00-0010, Accell SMARTpool, Mouse Tnpo3 (320938)) according to the manufacturers recommendations. 72 hours after adding siRNA, cells were washed and processed for western blotting or challenge with HIV as described.

### Owl monkey TRIMcyp fusion proteins (OMThNC and OMTmNC)

The Cyp domain of mouse and human Nup358 was PCR amplified from MEF cDNA and a human Nup358 full length plasmid respectively using the primers OMTRIMhNupCypS PshAI atatGACAGAAGTCcaacgctactgggacgccgccgcctgggaccttgtagcatcagc catgaatcctgtggtgttttttgatgtttgtgcggac, OMTRIMhNupCypAS NotI atatGCGGCCGCctatctgtccacattctgtgatagttattcttc, OMTRIMmNupCypS PshAI atatGACAGAAGTCcaacgctactgggacgccgccgcctgggaccttgtagcatcagccatgaatcctgtggtgttttttgatgtttgtgcggatgg and OMTRIMmNupCypAS NotI atatGCGGCCGCctagctgtccacattctgtgatacaaattcttc. The 553 base pair product was cloned into the PshA I and Not I sites of an owl monkey TRIMCyp (OMTC) encoding plasmid to create pOMThNup358Cyp (OM TRIM+human Nup358 Cyp) and pOMTmNup358Cyp (OM TRIM+mouse Nup358 Cyp). Dual promoter vectors expressing GFP and either HA-tagged OMTC, OMThNup358Cyp (abbreviated as OMThNC), or OMTmNup358Cyp (OMTmNC)were used to introduce the proteins into permissive Crandell feline kidney cells (CrFK). Cells were confirmed 100% GFP expressing by FACS using a BD Biosciences FACScan machine. Specific expression of fusion proteins as well as GFP was verified by western blot probed with rat anti-HA (3F10, Roche) at 1∶1000 and mAb against GFP (JL-8, Clontech) at 1∶8000 respectively. Tubulin was used as a loading control and detected with anti-alpha tubulin mAb (Sigma) at 1∶8000. Anti-rat secondary (Santa Cruz Biotechnology) at 1∶5000 and anti-mouse secondary (CalBiochem) at 1∶5000 were used correspondingly. Parental and engineered cells were challenged with HIV-1_luc_, HIV-1_luc_ with the N74D capsid mutation, FIVluc, and NB-MLVluc at two vector inputs. Luciferase activity was measured at 2 days post transduction and normalized to cell number.

Mouse CypA was amplified from MEF cDNA using primers ataggatccgccgccatggtcaaccccaccgtgttc and atatctcgagttagagctgtccacagtcggaaatggtgatc. This was inserted into a lentiviral vector where CypA expression was driven by an Ef1α promoter, and mCherry was expressed from an IRES. Human CypA was amplified from human cDNA, using the same 5′ primer and primer atatctcgagttaagcgtagtctgggacgtcgtatgggtattcgagttgtccacagtcagcaatggtgatc. Jurkat^PPIA−/−^ cells [44] were transduced with either human or mouse cyclophilin A lentiviral vectors, and FACS sorted for mCherry expression. WB using rabbit CypA antibody (Santa Cruz-133494) confirmed cyclophilin A expression.

### PCR for gene deletion

DNA was isolated from cells using DNeasy kit (Invitrogen) according to the manufacturers instructions. Primers and conditions for PCR were as described [58].

### Reporter virus and vector infections of stable −/− MEFs

20,000 cells were plated per well of a 24 well plate. For HIV-1 cells were challenged with HVI-1_luc_. HIV-1_luc_ is an HIV-1 NL4-3 reporter virus in which the viral accessory gene n*ef* was replaced with the firefly luciferase (*luc*) cDNA; it also has a 426 nt deletion in *env* [80]. The lowest input used for MEFs was 0.35 Reverse Transcriptase (RT) units per cell. Cells were washed the next day and harvested at 4–7 days post infection for luciferase activity using Bright Glo (Promega) as directed. Luciferase counts were normalized either to protein content or cell number. FIV luc, EIAV luc and MLV luc challenges were performed similarly.

### Growth arrest

Cells were cultured in aphidicolin 1 ug/ml for twenty four hours. Cells were then counted and plated for HIV-1 challenge as described (while maintained in aphidicolin), or processed for cell cycle analysis in the following manner. Cells were fixed in ice –cold 70% ethanol for 1 hour and then washed in PBS. Valkelovs Propidium iodide solution with RNAse A was added and incubated at 4°overnight. Cells were analyzed by FACS the next day.

### Cyclosporine assay

Five µM cyclosporine was added to indicated wells with challenge virus. The drug was washed off the next day and luciferase activity was analyzed as described above. Knockout Jurkat cells [44] were used as a negative control for cyclophilin western blotting.

### 2-LTR circles and viral integration

PCR for 2-LTR circles, total Gag and Alu-PCR was performed as described [83]. For 2-LTR circles cells were challenged with HIV-1 and harvested at 22 hours post transduction or at indicated time points. For integration, cells were transduced and passaged for 10 days before DNA was harvested and analyzed for total Gag copies. Both 2-LTR circles and Gag copies are normalized to GAPDH copies. Assay of HIV integrants in murine cells was performed as described, using the BBL-1 PCR assay [79].

## Supporting Information

Figure S1 **Susceptibility of wild type and G89V HIV-1 to Trim-Cyp protein inhibition.** OMTC: owl monkey TRIMCyp. OMhNC: protein in which the OMTC Cyp domain is replaced by the human Nup358 CHD.
(TIF)

Figure S2 **Nup358 knockout cell line analyses.** (**A**) PCR analysis of DNA isolated from Nup358^FF^ or Nup358^−/−[GFP1-1340]^ MEFS using primers spanning exon 2. The numbers below the lanes indicate individual F/F cell lines used. Expected bands are 650 bp for the Nup358 F locus and 120 bp for the Nup358 – locus. (**B**) Western blot analysis of same cell lines shown in A with rabbit Nup358 antibody. GFP-Nup358 is shown as a size control, it is bigger than Nup358 as expected. Tubulin is shown as loading control. (**C**) Flow cytometry for GFP fluorescence in control Nup358^F/F^ cells (red curves) and Nup358^−/−[GFP1-1340]^ cells (gray curves). Note the small shift to the right from the GFP1-1340 protein. For comparison, and as a positive control, the lower plot shows the GFP signal (green curve) after infection of Nup358^−/−[GFP1-1340]^ cells with a GFP-encoding HIV-1 vector. Consistent with these results and with the observations of Hamada et al. [58], GFP1-1340 is not visible by standard epifluorescence microscopy in Nup358^−/−[GFP1-1340]^ cells (data not shown).
(TIF)

Figure S3 **Growth curves of indicated cell lines.** 17 and 18 refer to independently derived MEF cell lines.
(TIF)

Figure S4 **2-LTR circle analysis in indicated cell lines, normalized to GAPDH copies.**
(TIF)

Figure S5 **Representative propidium iodide FACS analysis of MEF cells either cycling (top graph) or after growth arrest with aphidicolin 1 µg/ml for 24 hours (lower graph).** The 18^FF^ cells are shown here.
(TIF)

Figure S6 **Alignment of human and murine Nup358Cyp domains.**
(TIF)

Figure S7 **PCR analysis of genomic DNA isolated from indicated cell lines, using primers that span exon 2.** Expected bands are 650 bp for the Nup358 F locus and 120 bp for the Nup358 – locus.
(TIF)

Figure S8 **Analysis of effects of GFP1-3224 complementation of −/− cells.** (**A**) Indicated cell lines were challenged with a range of HIV-1_luc_ dilutions. (**B**) Immunoblotting. Equal numbers of cells from 18^−/−[GFP1-3224]^ and 18^F/F^ MEF lines were harvested and used for western blots using antibodies to Nup358 and tubulin. Two different volumes of the same cell lysates were electrophoresed. GFP1-3224 (lane 1) is relatively over-expressed compared to the endogenous levels of Nup358 (lane 2).
(TIF)

Figure S9 **Comparison of WT and G89A HIV-1 vectors in SupT1 cells, with or without acute Nup358 knockdown with shRNA-encoding vectors.** HIV infections were carried out 96 hours after shRNA transduction as in Figure 8. WT and G89A vectors were prepared in parallel and inputs were RT activity unit-normalized. Intracellular luciferase activities were measured 72 hours after infection. Two independent experiments are shown. The reason for the flatter dose-response slope in Nup358-depleted cells in the second experiment is unknown.
(TIF)

Figure S10 **Challenge of SupT1 cells with luciferase encoding retroviral vectors.** Infections labeled acute were done six days after knockdown with lentiviral vector encoding shRNA and mCherry and cells were uniformly mCherry-positive. The stable cells are described in text and legend for Figure 8D.
(TIF)
