# Supplementary figures and images for: A Cyclophilin Homology Domain-Independent Role for Nup358 in HIV-1 Infection
Source: PLoS Pathog. 2014 Feb 20;10(2):e1003969. doi: 10.1371/journal.ppat.1003969 (PMC3930637; doi:10.1371/journal.ppat.1003969)

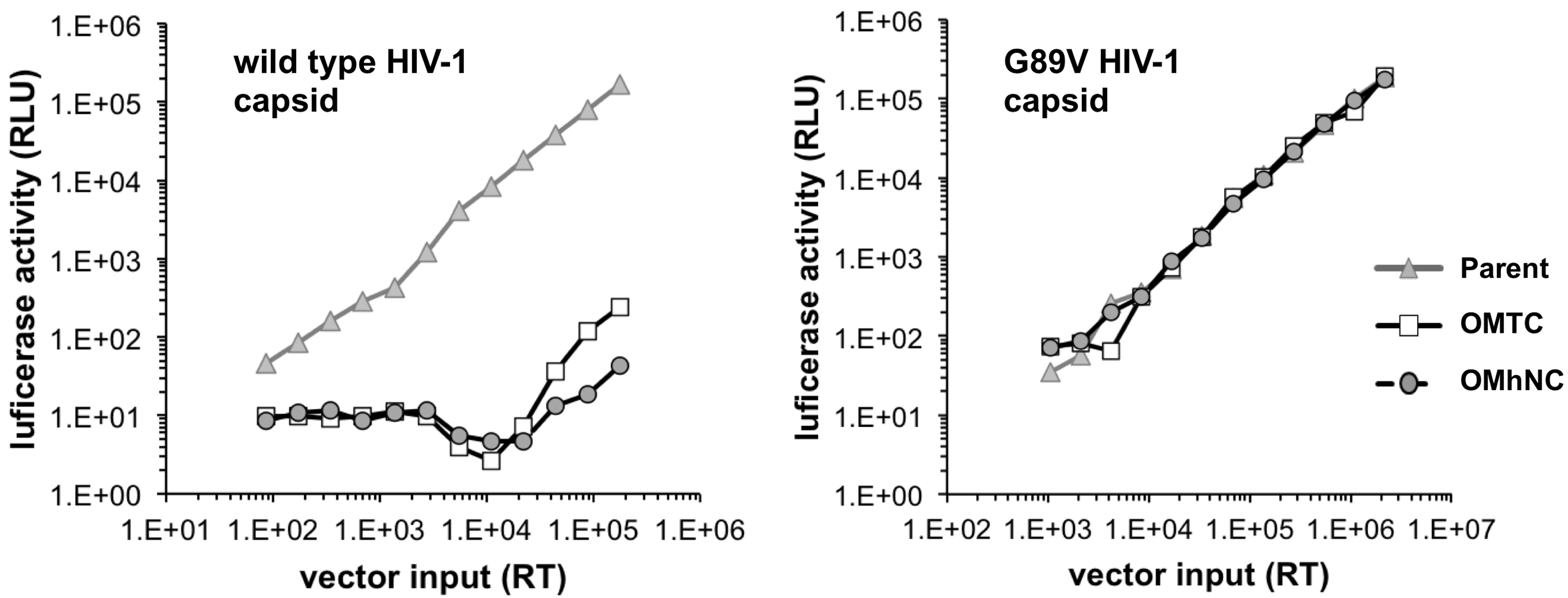

Supplement: Figure S1 — Susceptibility of wild type and G89V HIV-1 to Trim-Cyp protein inhibition. OMTC: owl monkey TRIMCyp. OMhNC: protein in which the OMTC Cyp domain is replaced by the human Nup358 CHD. (TIF) [file ppat.1003969.s001.tif]

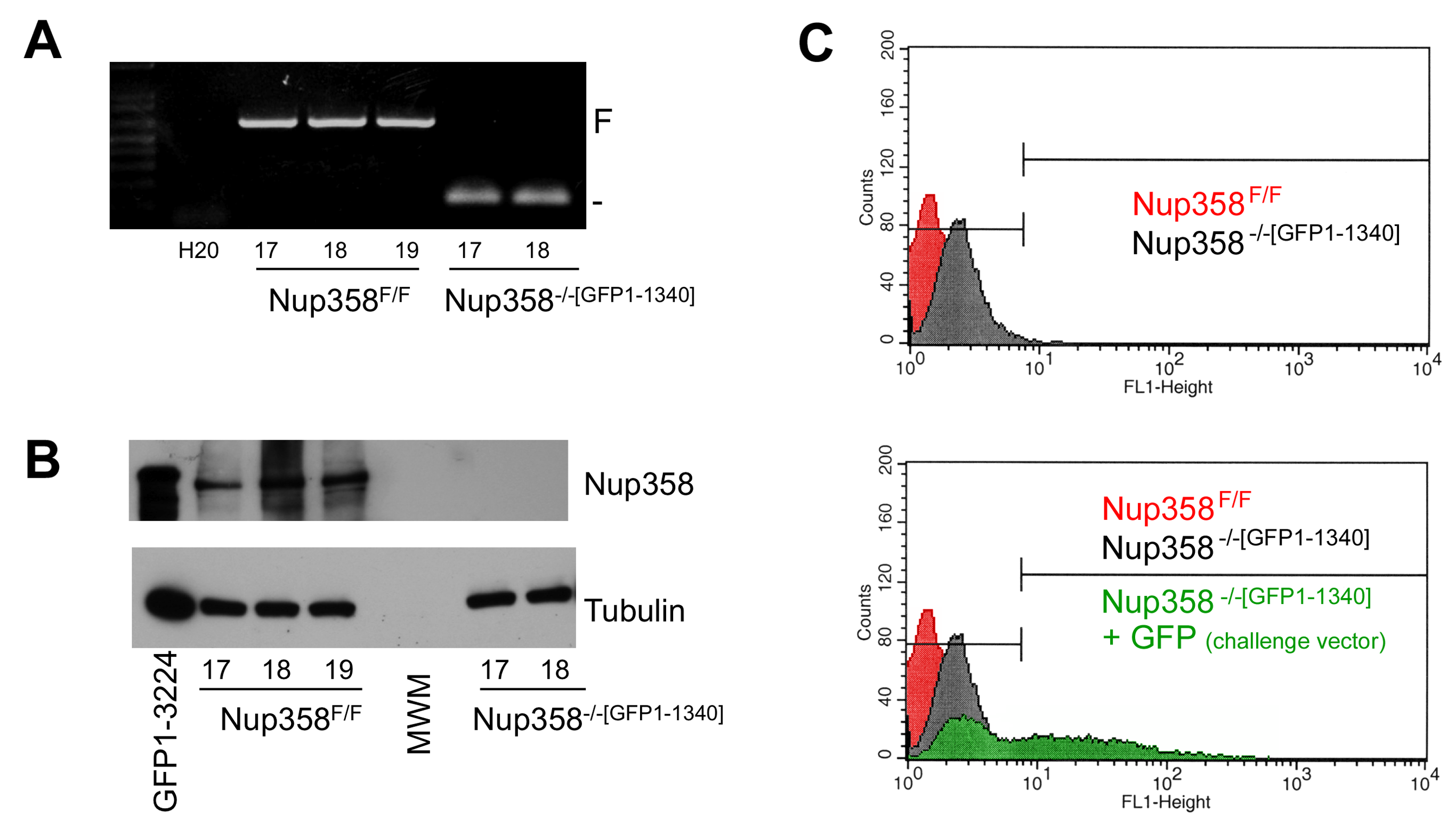

Supplement: Figure S2 — Nup358 knockout cell line analyses. (A) PCR analysis of DNA isolated from Nup358FF or Nup358−/−[GFP1-1340] MEFS using primers spanning exon 2. The numbers below the lanes indicate individual F/F cell lines used. Expected bands are 650 bp for the Nup358 F locus and 120 bp for the Nup358 – locus. (B) Western blot analysis of same cell lines shown in A with rabbit Nup358 antibody. GFP-Nup358 is shown as a size control, it is bigger than Nup358 as expected. Tubulin is shown as loading control. (C) Flow cytometry for GFP fluorescence in control Nup358F/F cells (red curves) and Nup358−/−[GFP1-1340] cells (gray curves). Note the small shift to the right from the GFP1-1340 protein. For comparison, and as a positive control, the lower plot shows the GFP signal (green curve) after infection of Nup358−/−[GFP1-1340] cells with a GFP-encoding HIV-1 vector. Consistent with these results and with the observations of Hamada et al. [58], GFP1-1340 is not visible by standard epifluorescence microscopy in Nup358−/−[GFP1-1340] cells (data not shown). (TIF) [file ppat.1003969.s002.tif]

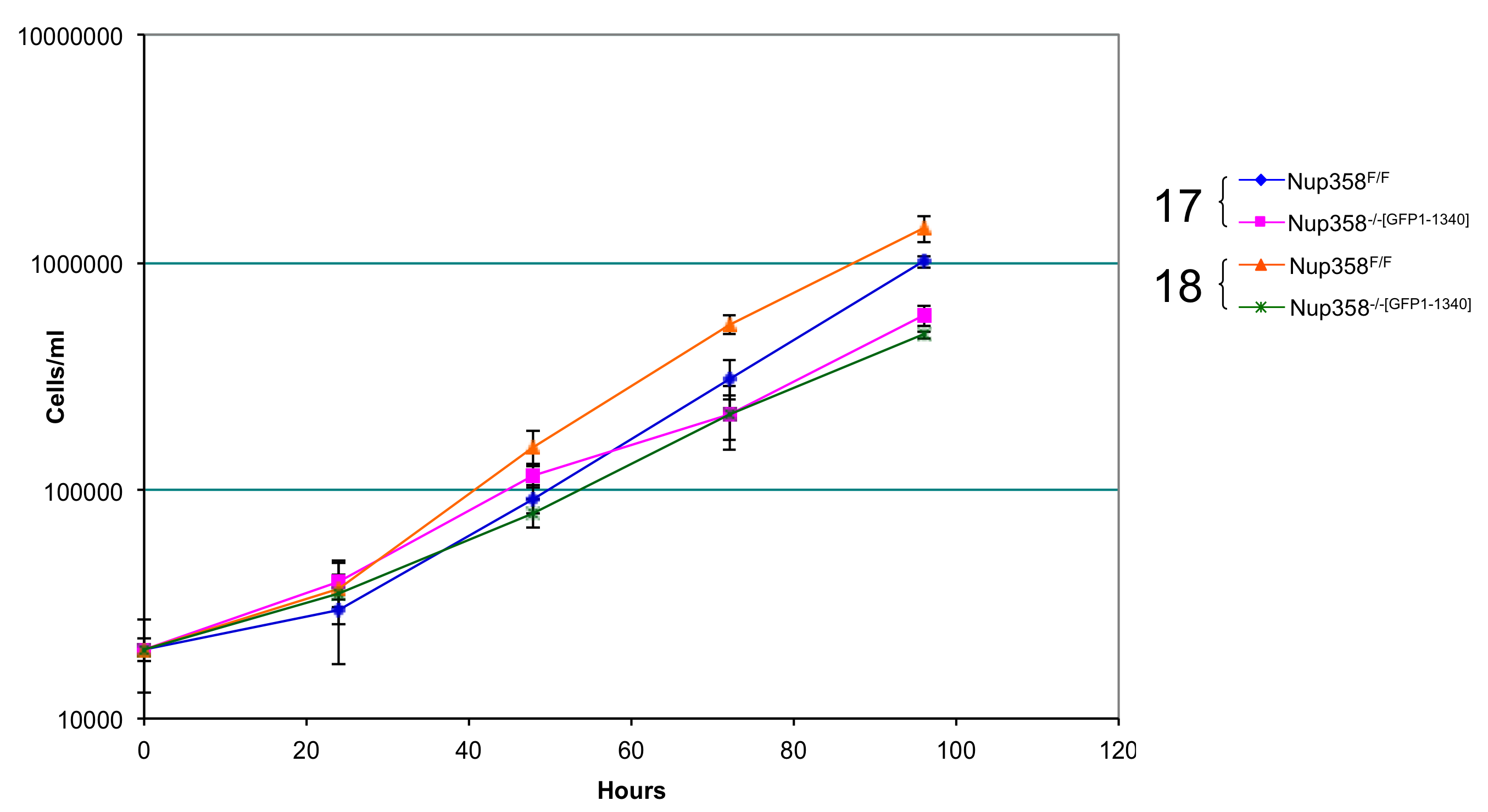

Supplement: Figure S3 — Growth curves of indicated cell lines. 17 and 18 refer to independently derived MEF cell lines. (TIF) [file ppat.1003969.s003.tif]

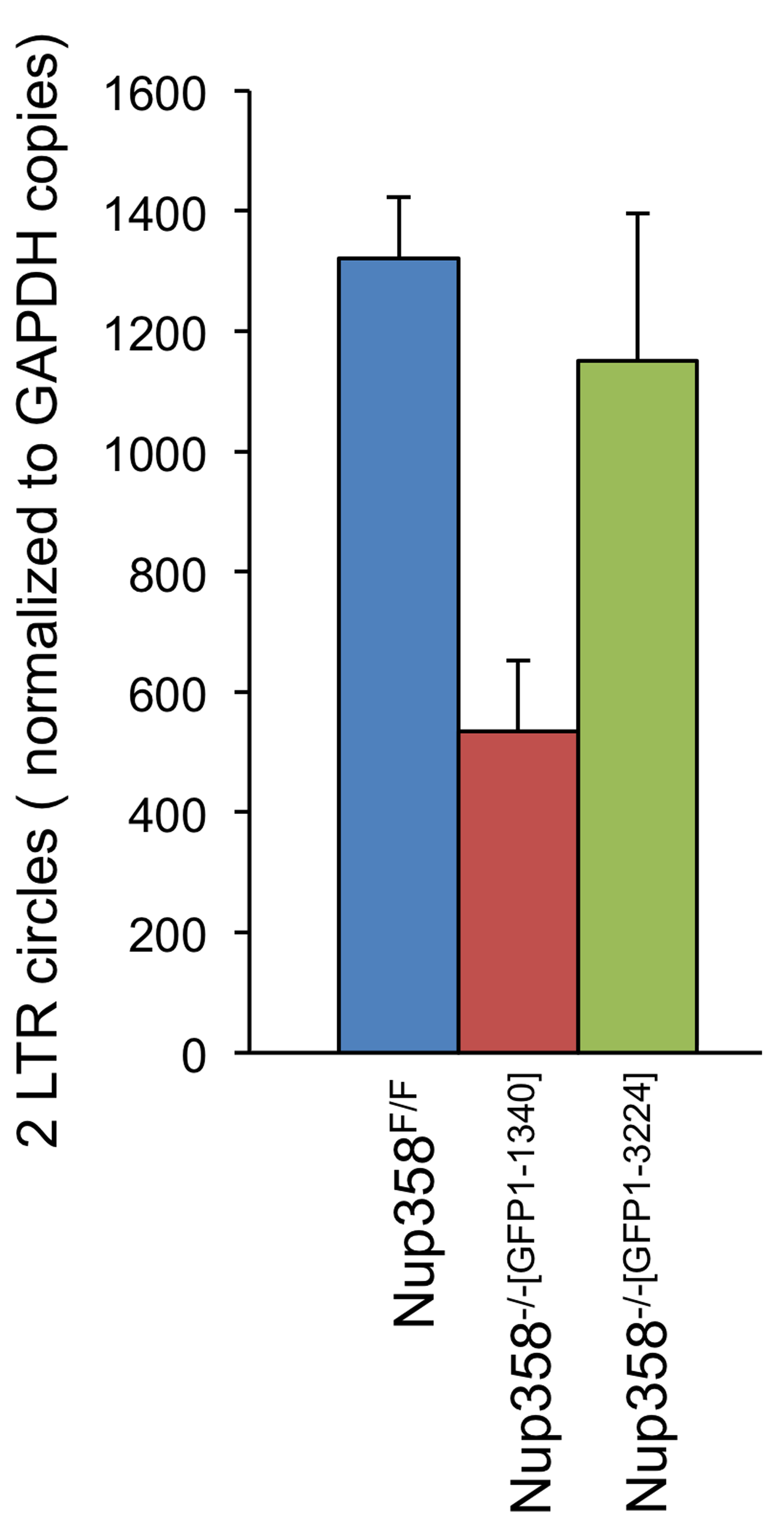

Supplement: Figure S4 — 2-LTR circle analysis in indicated cell lines, normalized to GAPDH copies. (TIF) [file ppat.1003969.s004.tif]

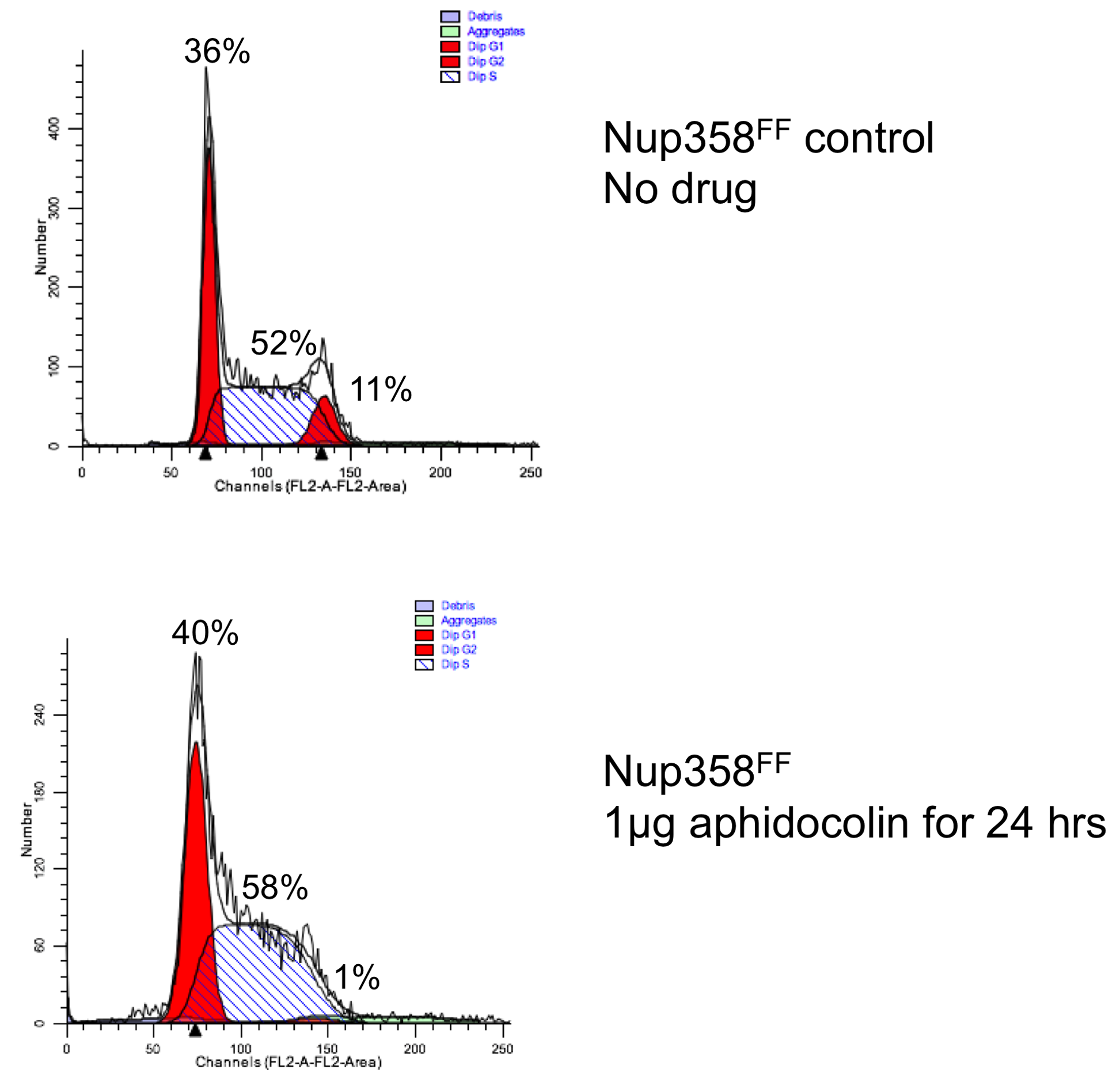

Supplement: Figure S5 — Representative propidium iodide FACS analysis of MEF cells either cycling (top graph) or after growth arrest with aphidicolin 1 µg/ml for 24 hours (lower graph). The 18FF cells are shown here. (TIF) [file ppat.1003969.s005.tif]

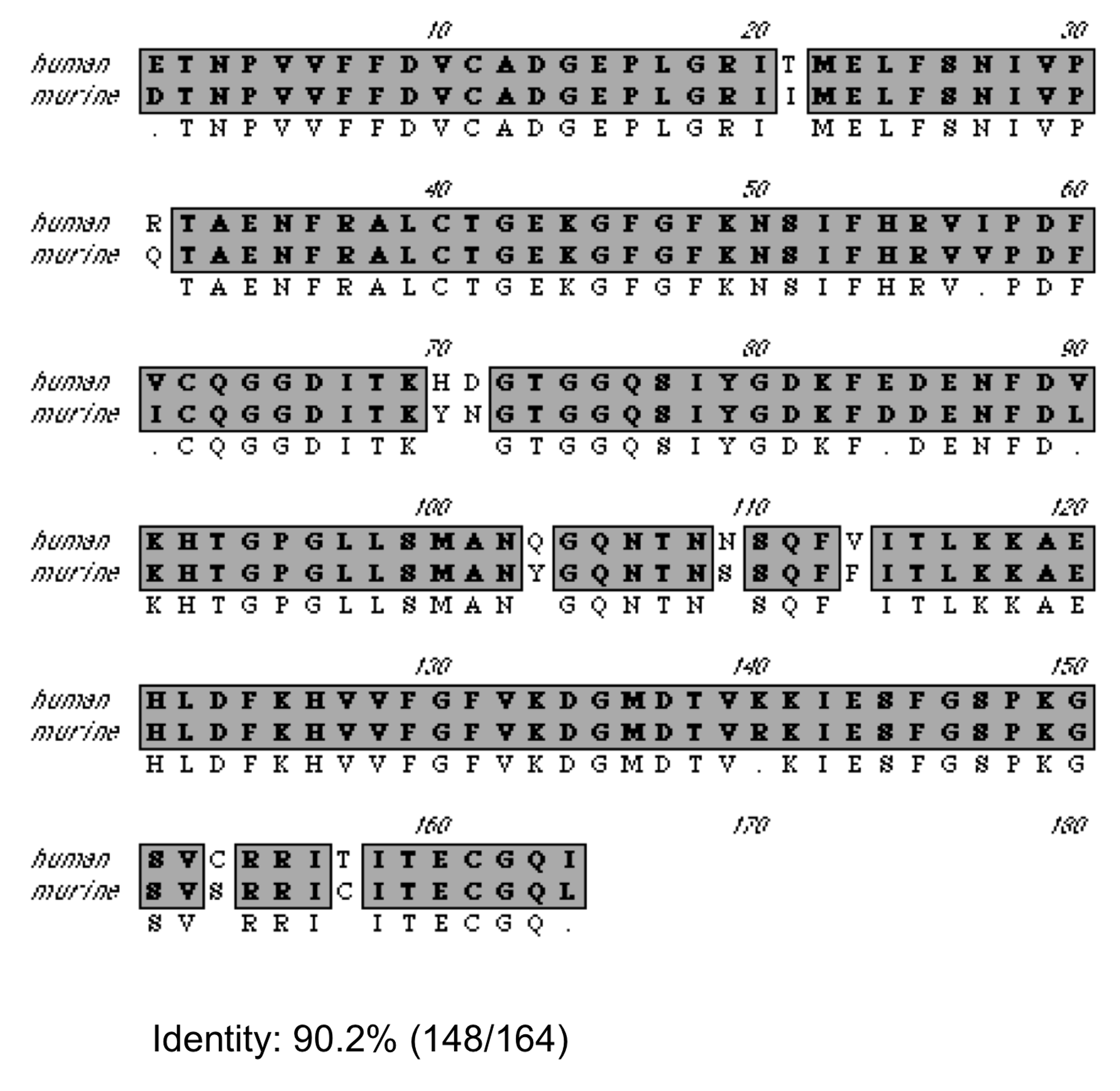

Supplement: Figure S6 — Alignment of human and murine Nup358Cyp domains. (TIF) [file ppat.1003969.s006.tif]

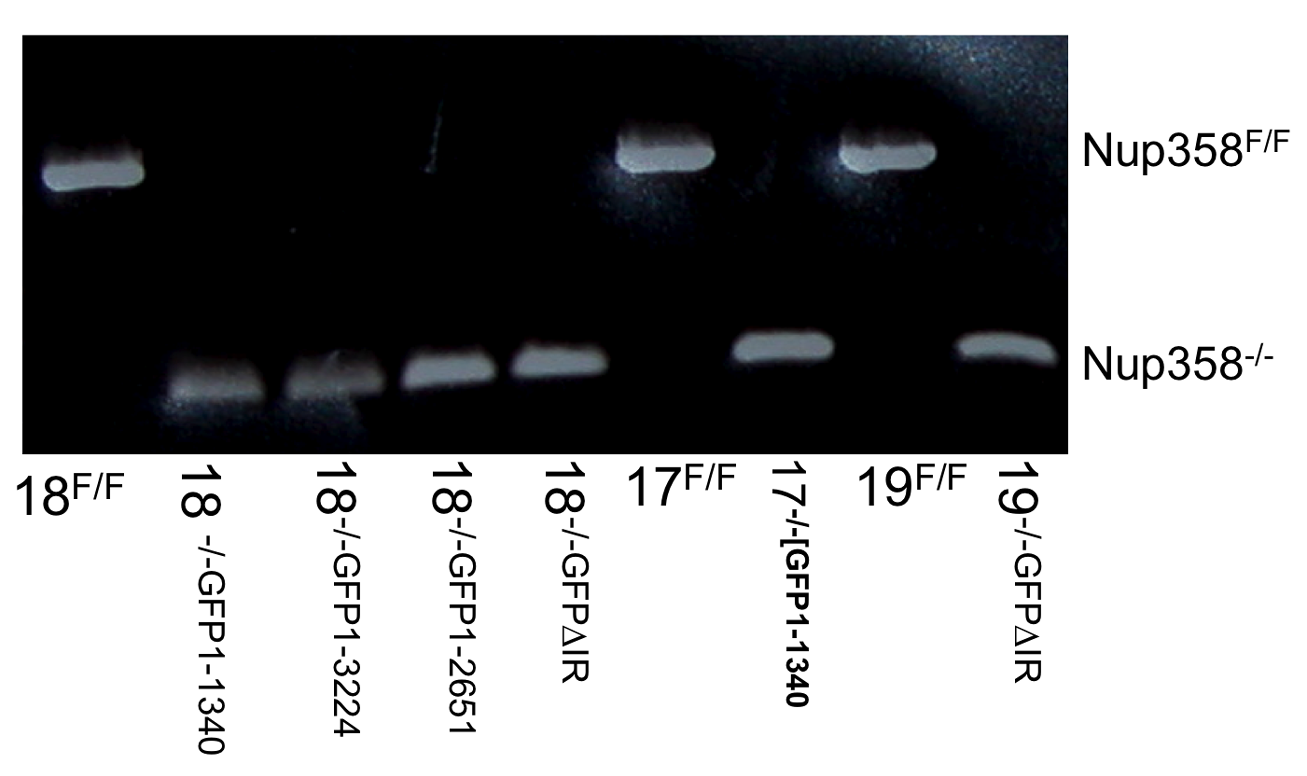

Supplement: Figure S7 — PCR analysis of genomic DNA isolated from indicated cell lines, using primers that span exon 2. Expected bands are 650 bp for the Nup358 F locus and 120 bp for the Nup358 – locus. (TIF) [file ppat.1003969.s007.tif]

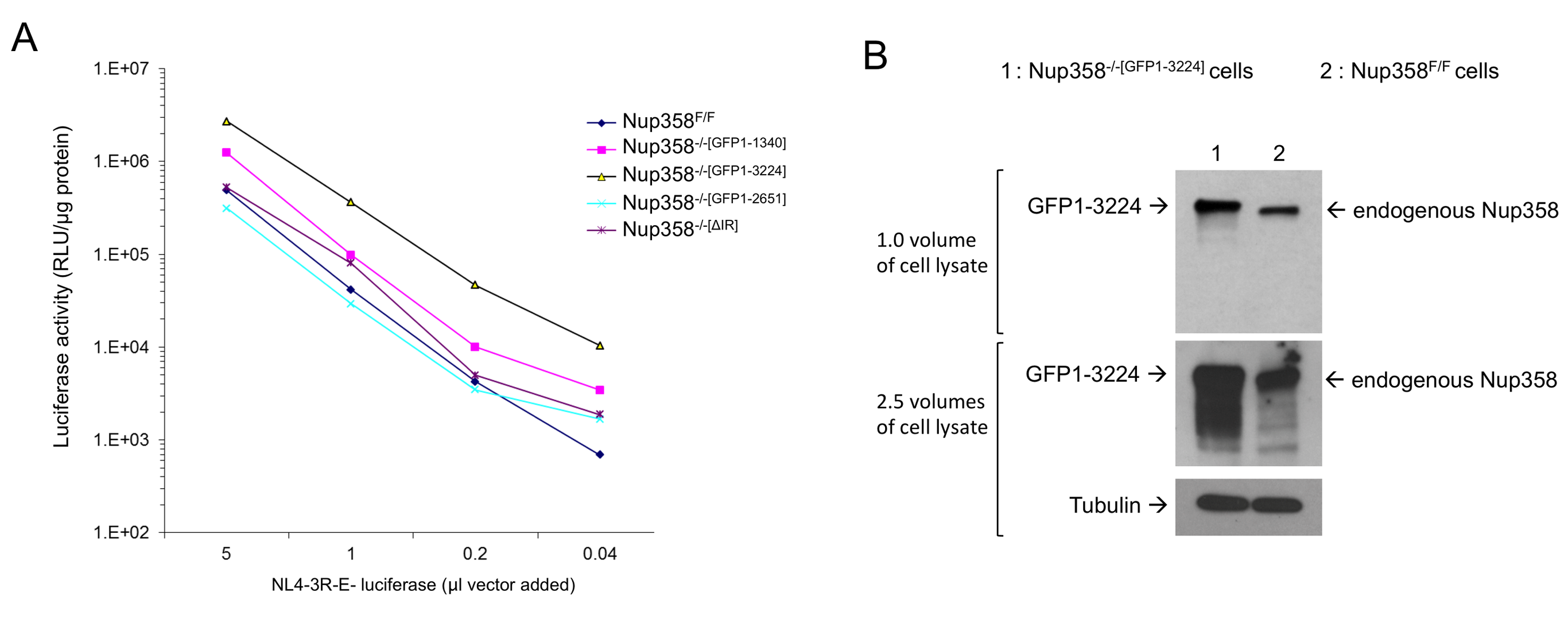

Supplement: Figure S8 — Analysis of effects of GFP1-3224 complementation of −/− cells. (A) Indicated cell lines were challenged with a range of HIV-1luc dilutions. (B) Immunoblotting. Equal numbers of cells from 18−/−[GFP1-3224] and 18F/F MEF lines were harvested and used for western blots using antibodies to Nup358 and tubulin. Two different volumes of the same cell lysates were electrophoresed. GFP1-3224 (lane 1) is relatively over-expressed compared to the endogenous levels of Nup358 (lane 2). (TIF) [file ppat.1003969.s008.tif]

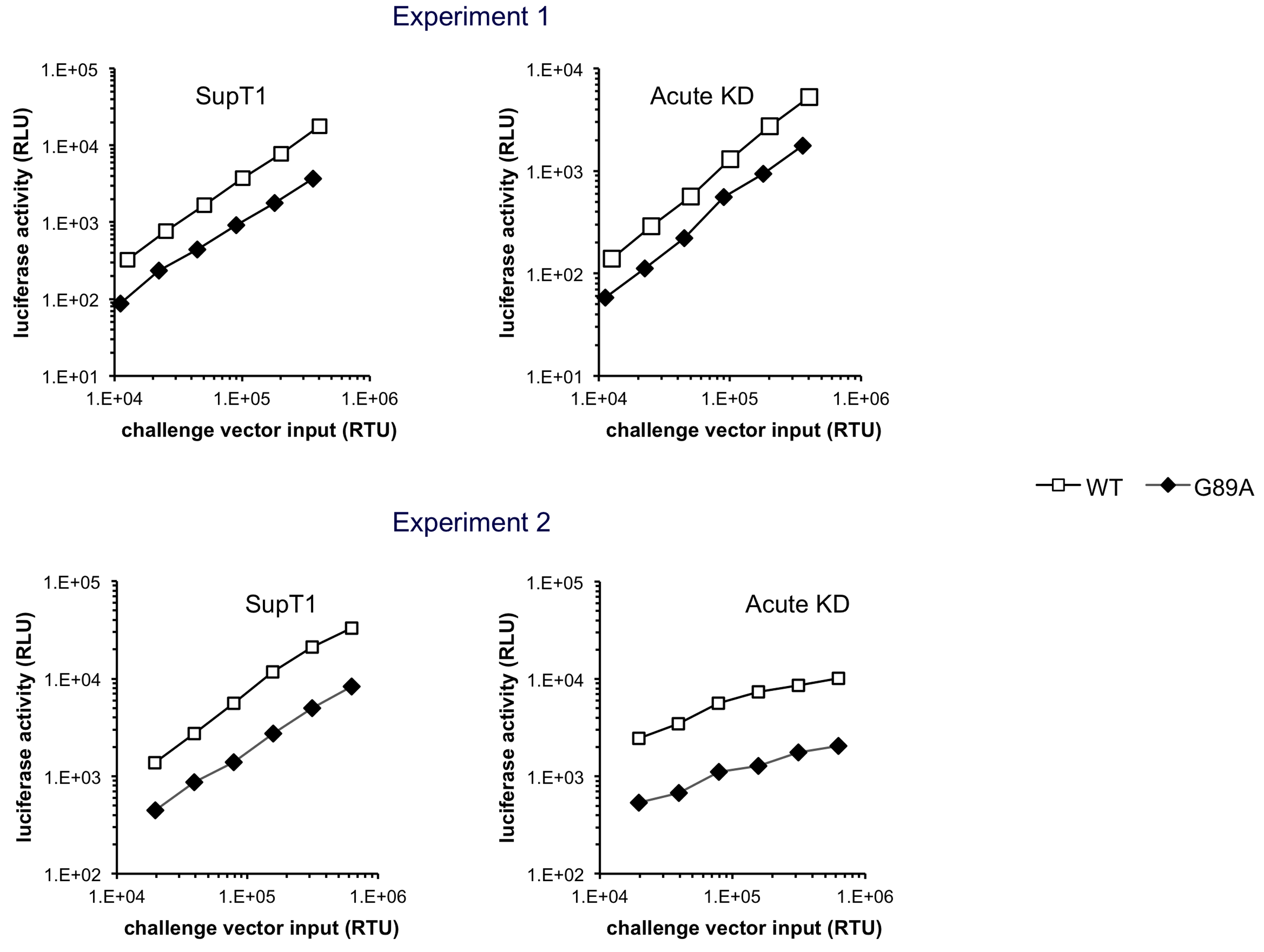

Supplement: Figure S9 — Comparison of WT and G89A HIV-1 vectors in SupT1 cells, with or without acute Nup358 knockdown with shRNA-encoding vectors. HIV infections were carried out 96 hours after shRNA transduction as in Figure 8. WT and G89A vectors were prepared in parallel and inputs were RT activity unit-normalized. Intracellular luciferase activities were measured 72 hours after infection. Two independent experiments are shown. The reason for the flatter dose-response slope in Nup358-depleted cells in the second experiment is unknown. (TIF) [file ppat.1003969.s009.tif]

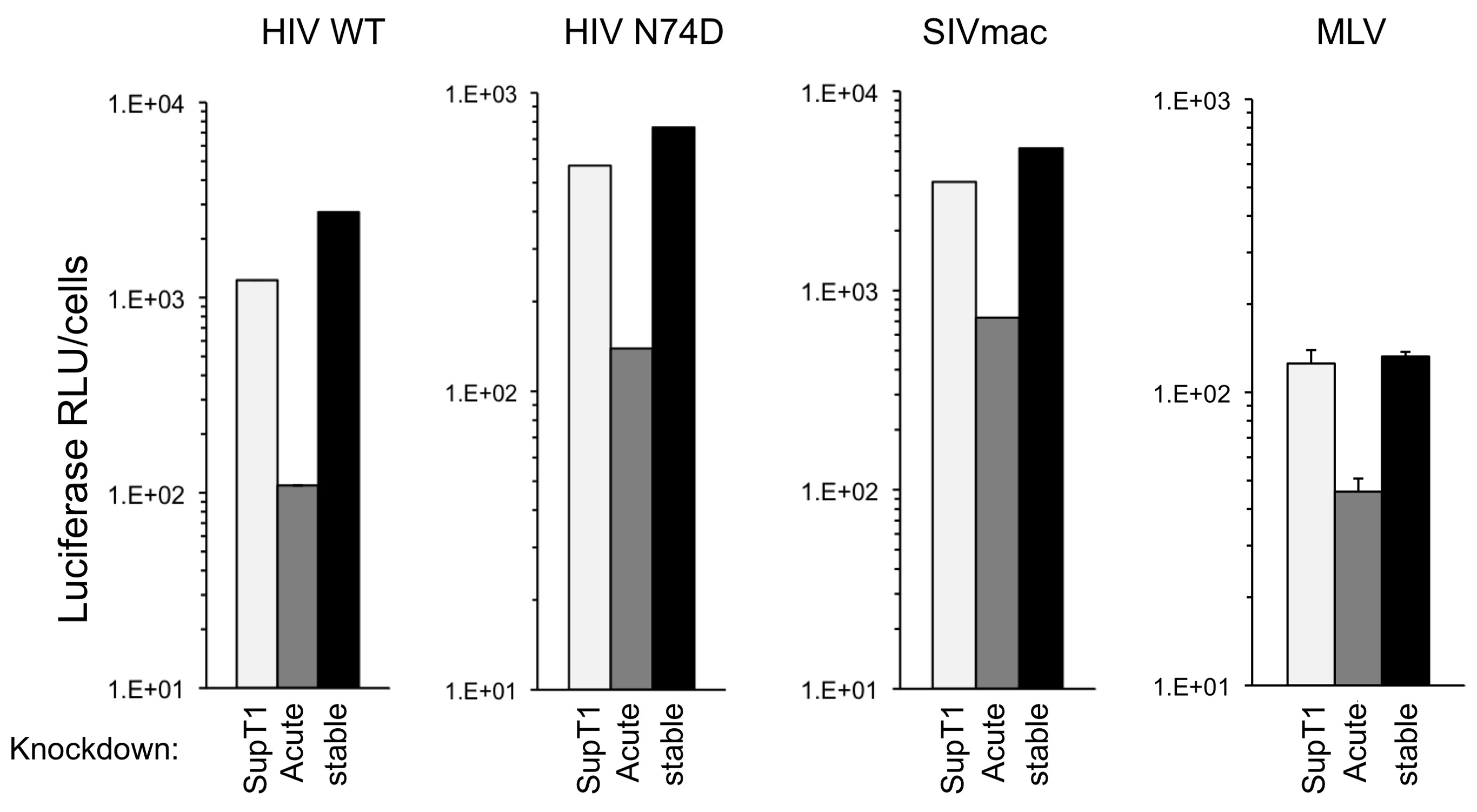

Supplement: Figure S10 — Challenge of SupT1 cells with luciferase encoding retroviral vectors. Infections labeled acute were done six days after knockdown with lentiviral vector encoding shRNA and mCherry and cells were uniformly mCherry-positive. The stable cells are described in text and legend for Figure 8D. (TIF) [file ppat.1003969.s010.tif]
